# Heme Spin Distribution in the Substrate-Free and Inhibited Novel CYP116B5hd: A Multifrequency Hyperfine Sublevel Correlation (HYSCORE) Study

**DOI:** 10.3390/molecules29020518

**Published:** 2024-01-20

**Authors:** Antonino Famulari, Danilo Correddu, Giovanna Di Nardo, Gianfranco Gilardi, George Mitrikas, Mario Chiesa, Inés García-Rubio

**Affiliations:** 1 Departamento de Física de la Materia Condensada, Universidad de Zaragoza, C/Pedro Cerbuna 12, 50009 Zaragoza, Spain; tonyfamulari@unizar.es; 2 Department of Chemistry, University of Turin, Via Giuria 9, 10125 Torino, Italy; mario.chiesa@unito.it; 3 Department of Life Sciences and Systems Biology, University of Turin, Via Accademia Albertina 13, 10123 Torino, Italygiovanna.dinardo@unito.it (G.D.N.); gianfranco.gilardi@unito.it (G.G.); 4 Institute of Nanoscience and Nanotechnology, NCSR Demokritos, 15341 Athens, Greece; g.mitrikas@inn.demokritos.gr; 5 Instituto de Nanociencia y Materiales de Aragón (INMA), CSIC-Universidad de Zaragoza, 50009 Zaragoza, Spain

**Keywords:** EPR spectroscopy, CYP450, HYSCORE, peroxygenase, hyperfine interactions, low-spin hemeprotein, multifrequency EPR, quadrupole interaction, imidazole binding

## Abstract

The cytochrome P450 family consists of ubiquitous monooxygenases with the potential to perform a wide variety of catalytic applications. Among the members of this family, CYP116B5hd shows a very prominent resistance to peracid damage, a property that makes it a promising tool for fine chemical synthesis using the peroxide shunt. In this meticulous study, we use hyperfine spectroscopy with a multifrequency approach (X- and Q-band) to characterize in detail the electronic structure of the heme iron of CYP116B5hd in the resting state, which provides structural details about its active site. The hyperfine dipole–dipole interaction between the electron and proton nuclear spins allows for the locating of two different protons from the coordinated water and a beta proton from the cysteine axial ligand of heme iron with respect to the magnetic axes centered on the iron. Additionally, since new anti-cancer therapies target the inhibition of P450s, here we use the CYP116B5hd system—imidazole as a model for studying cytochrome P450 inhibition by an azo compound. The effects of the inhibition of protein by imidazole in the active-site geometry and electron spin distribution are presented. The binding of imidazole to CYP116B5hd results in an imidazole–nitrogen axial coordination and a low-spin heme Fe^III^. HYSCORE experiments were used to detect the hyperfine interactions. The combined interpretation of the gyromagnetic tensor and the hyperfine and quadrupole tensors of magnetic nuclei coupled to the iron electron spin allowed us to obtain a precise picture of the active-site geometry, including the orientation of the semi-occupied orbitals and magnetic axes, which coincide with the porphyrin N-Fe-N axes. The electronic structure of the iron does not seem to be affected by imidazole binding. Two different possible coordination geometries of the axial imidazole were observed. The angles between *g_x_* (coinciding with one of the N-Fe-N axes) and the projection of the imidazole plane on the heme were determined to be −60° and −25° for each of the two possibilities via measurement of the hyperfine structure of the axially coordinated ^14^N.

## 1. Introduction

The cytochrome P450 family (CYP450s) consists of ubiquitous and versatile monooxygenases primarily responsible for catalyzing the hydroxylation of non-activated hydrocarbons using O_2_ and NADPH, but they are also involved in numerous other reactions [1]. Existing in all living organisms such as human beings, animals, plants, bacteria, and fungi, CYP450s catalyze many important biological processes [2,3]. This great catalytic versatility, exploited in different ways to obtain high-value products such as steroids, fatty acids, and prostaglandins, as well as to eliminate xenobiotics and drug metabolites [4,5,6] has, over the years, attracted much interest in the scientific community. Additionally, CYP450s have also found use in the catalysis of specific reactions, such as epoxidation, desaturation, O, S, N-dealkylation, and sulfoxidation, as well as, if conveniently engineered, unnatural reactions [7,8,9,10,11,12]. Lately, CYP450s have also been reported to be key enzymes in cancer generation and treatment because they mediate the metabolic activation of numerous precarcinogens and participate in the inactivation and activation of anticancer drugs [13]. In this sense, azo compounds are part of several inhibitor-based drugs targeting CYP450s [14,15]. Finally, unwanted inhibition of CYP450s during drug metabolism and transport is one of the main causes of drug–drug interactions (DDIs) with consequent hospitalization and deaths related to drug use [16]. For these reasons, developing therapies allowing the possibility of controlling CYP450 inhibition is very attractive and could lead to substantial improvement in the treatment of certain medical conditions.

Among the members of the wide CYP450 family, CYP116B5 belongs to class VII CYP450 [17], which is defined as “self-sufficient” CYP450s [18] because they possess the P450 domain fused with the CYP450 reductase domain. Low activity, poor stability, and cofactor dependence are the main barriers to the industrial/biotechnological applicability of CYP450s and limit their use only to the production of high-value molecules such as fine chemicals and pharmaceuticals. Therefore, various efforts have been undertaken in recent decades to increase the application of CYP450s in biotechnology owing to enzyme engineering. For example, the heme domain (CYP116B5hd) obtained from the full protein CY116B5 showed outstanding resistance to H_2_O_2_ damage. This feature allows the enzyme to use the so-called peroxide shunt path to perform catalysis and, therefore, carry out different oxidative reactions on aromatic compounds and generate drug metabolites without economic or environmental drawbacks [19]. This pathway is a shortcut of the CYP450 catalytic cycle consisting in the use of hydrogen peroxide or peracids to generate, directly from the resting state, Compound 0, which precedes the formation of reactive Compound I, the true catalytic species of the whole catalytic cycle. Then, the latter can, through a stepwise or dynamically concerted radical rebound mechanism, immediately perform the hydroxylation reaction [20,21]. Therefore, without needing expensive electron donors, such as NADPH, used in the classical CYP450 catalysis, but simply by using hydrogen peroxide or peracids, products can be obtained with high catalytic performances [19,22]. 

The exceptional behavior of CYP116B5hd, which makes it different from a classical CYP450 monooxygenase, relies on the potential to exploit its peroxygenase-like reactivity [23]. With the conviction that this particular behavior had to be investigated by focusing on the properties and characteristics of the CYP116B5hd active site—the place where all the catalytic events take place—we undertook the study of the resting state of the enzyme via electron paramagnetic resonance (EPR) spectroscopy. Since CYP450s possess a characteristic active site with an Fe^III^-heme center as the fulcrum, and given the paramagnetic nature of the iron, not only in the resting state of the enzyme but also in several intermediates generated during the CYP450 catalytical cycle, EPR spectroscopy lends itself as a very suitable technique to characterize and analyze this system [24,25,26,27,28,29,30,31]. In our previous work [32,33], we carried out a study on CYP116B5hd, where we analyzed the ***g***-values according to the electronic model for low-spin Fe^III^ by Taylor [34,35] and compared our results with those obtained for other classical (monooxygenase-like) CYP450s, such as CYP102A1 (CYPBM3), and more exotic (peroxygenase-like) CYP450s, such as CYP152B1, CYP152K6, and CYP152L1. From this comparison, it was concluded that the electronic state of the enzyme was very much like the classic P450, determined by the active site in the close proximity of iron. Therefore, the peculiar behavior of this enzyme with respect to peroxide damage resistance would rather be associated with the supramolecular interactions between the protein scaffold and its active site. In the same study, the inhibitor imidazole was shown to bind the protein at the active site through direct coordination with the heme iron. The proof was the finding of an imidazole ^14^N nucleus coupled with the electron spin of the iron using hyperfine sublevel correlation (HYSCORE) experiments. However, the complete analysis of nuclear spin frequencies obtained via HYSCORE spectroscopy or, in general, via hyperfine spectroscopy can still expose a wealth of information encoded in the hyperfine and nuclear quadrupole interactions. Such information is related to the geometry of the active site; for example, the location of a magnetic nucleus in virtue of its through-space magnetic interaction with the electron spin or related to the mapping of the electron spin density distribution [36,37,38,39,40,41]. This knowledge is relevant to enable structure–function relationships and to develop a molecular-level understanding of the factors governing the catalytic properties of heme-based enzymes.

This work, in particular, aims to clarify the coordination of the axial water molecule, since water molecules connected to the active site can shuttle proton channels and influence reactivity [42,43]. They definitely play an active role during the catalytic cycle of CYP450s, whether they electronically stabilize the Fe^III^ center in the resting state, favor the substrate approach to the active site, or the protonation of catalytic intermediates [44,45,46]. At the same time, there is the goal of conveying the geometric details of the active site and, especially, the orientation of the third iron orbitals where the unpaired electron resides and relating it with the coordination of the axial water molecule to find out the possible structural determinants of the electron distribution. Finally, the EPR spectroscopy characterization of CYP116B5hd is intended to be used as a model to study CYP450 inhibition by azo compounds and the effects of imidazole coordination on the structure of the heme. 

In order to access this information, the complete hyperfine and nuclear quadrupole tensors have to be determined experimentally. We conducted this determination in a disordered sample (protein frozen solution) by taking advantage of the anisotropic ***g***-tensor and systematically performing mostly HYSCORE experiments at different magnetic field values through the EPR spectrum, with special interest in the magnetic field positions that contain a principal axis of the ***g***-tensor. Starting from there, we report on a detailed multifrequency (X-band and Q-band microwave frequencies) EPR spectroscopy investigation of the heme iron properties of CYP116B5hd, a self-sufficient monooxygenase acting as a peroxygenase, either in its resting state or interacting with imidazole, a basic model for azo compounds. This study was carried out by means of CW-EPR combined with hyperfine spectroscopy, providing a precise description of the electronic structure and environment of this peculiar cytochrome P450. 

## 2. Results and Analysis

### 2.1. Effect of Nuclear Spin Labeling on the CW-EPR Spectrum 

The CW-EPR spectrum of CYP116B5hd as a substrate-free protein either in an aqueous or deuterated frozen solution showed the typical powder pattern of an Fe^III^ low-spin (*S* = ½) heme center (see Figure S1 in Supplementary Materials) with ***g***-values (see Table 1) coinciding, within the error limits, with the ones previously reported [32]. 

In the presence of an excess of imidazole or ^15^N_2_-imidazole, the CW-EPR spectrum is characterized by two new low-spin populations quantified to be approximately in the same amount, with neither one retaining the substrate-free protein ***g***-values, as was found before in the protein–imidazole complex [32]. 

Labeling the nuclei of the axial distal ligand, H_2_O or imidazole, with isotopes ^2^H and ^15^N, did not have any effect on the CW-EPR spectra, and consequently, on the crystal field parameters Δ and *V*. Therefore, one can conclude that the hyperfine couplings of the iron with these nuclei are smaller than other line-broadening mechanisms. To access the information about the hyperfine interactions occurring between the iron spin and the magnetic nuclei present in its environment, we previously tested the suitability of HYSCORE experiments, which showed couplings with several nuclei in the active site of the protein [32,33]. To fully characterize the anisotropy of the hyperfine and nuclear quadrupole interactions, HYSCORE experiments were recorded at different values of the magnetic field spanning the whole EPR spectrum of the protein, both with and without imidazole. To minimize the effect of blind spots, several τ values were used for the most significative spectra.

### 2.2. Hyperfine Interactions with Hydrogen Nuclei

The information related to couplings with hydrogen nuclei is observable only in the (+,+) quadrant, suggesting that these nuclei are weakly coupled to the iron electron spin. Among the closest protons to the iron are those belonging to the axial ligands, i.e., the two protons of the distal water molecule and the β (closest) or α protons of the proximal cysteine ligand (a.a. 381), and then the meso and pyrrole protons in the porphyrin ring.

**Figure 1:**
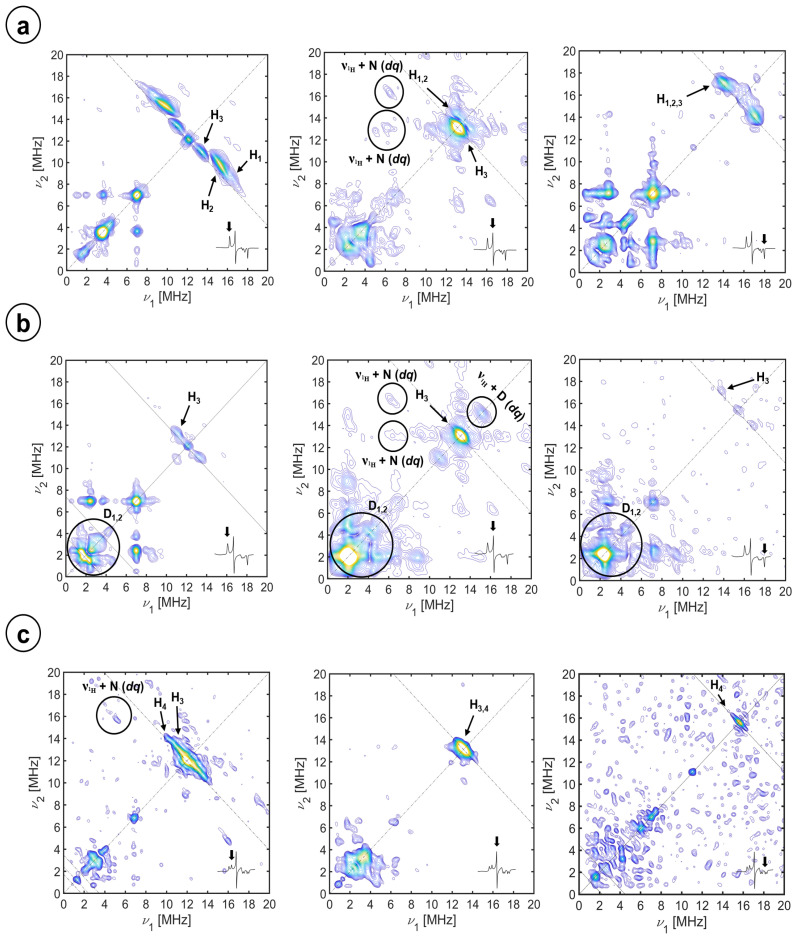
X-band, (+,+) quadrant, HYSCORE spectra of CYP116B5hd (300 μM) in KPi 50 mM pH 6.8, 30% glycerol substrate-free in H_2_O (**a**), substrate-free in D_2_O (**b**), and interacting with ^15^N-imidazole (**c**). The spectra, as shown in the insets, were recorded at the (left column) *g*_z_, (center column) *g*_y_, and (right column) *g*_x_ magnetic field positions. The spectra were recorded at 10 K. τ values of (**a**) the sum of 208 ns and 250 ns spectra, 250 ns and 250 ns; (**b**) 400 ns, 250 ns, and 250 ns; and (**c**) the sum of 208 ns and 250 ns spectra, 250 ns and 168 ns.

In Figure 1, the (+,+) quadrants of the HYSCORE spectra best depicting the proton signals at the magnetic field position corresponding to the principal magnetic axes are shown. 

All the proton signals emerge in the spectra as small ridges symmetrically placed with respect to the diagonal of the quadrant and located at or slightly above the antidiagonal crossing at the proton Larmor frequency for every magnetic field (dashed lines in the figure), consistent with proton signals in the weak-coupling regimen (A < 2ν_H_). The lower range of frequencies in the 2D spectrum shows signals attributed to the ^14^N nuclei, which are analyzed in the next section. The upper row of the figure (Figure 1a) collects the spectra from the resting state of the enzyme in an aqueous solution at the magnetic field positions corresponding to the *g_z_* (left spectrum), *g_y_* (center), and *g_x_* (right). 

For the *g_z_* spectrum, three short ridges with slightly different directions are detected; they have been assigned to three different protons in the heme environment and labeled H_1_, appearing at (15.9, 9.3) MHz, H_2_ at (15.5, 9.5) MHz, and H_3_ at (13.5, 10.9) MHz. It has been reported for CYP450s and other heme enzymes that the principal axis *g_z_* is oriented approximately along the heme normal plane [47]. This means that the external magnetic field is perpendicular to the heme plane for this single-crystal position spectrum, which is an excellent opportunity to assign the proton signals using the electron–nuclear dipole–dipole interaction (Equation (1)). This approximation should be accurate enough when the interacting nucleus is located more than 0.25 nm away from the metal center and there is no significant spin delocalization out of the iron ion (see page 30 of [48]):

(1) $$A_{\mathrm{dip}}=\frac{\mu_{0}}{4\pi h}{\;g}_{e\;}\mu_{B}g_{N\;}\mu_{N}\frac{\left(\;3\cos^{2}\theta -1\right)}{r^{3}}=T\;(3\cos^{2}\theta -\;1)$$

**Figure 2:**
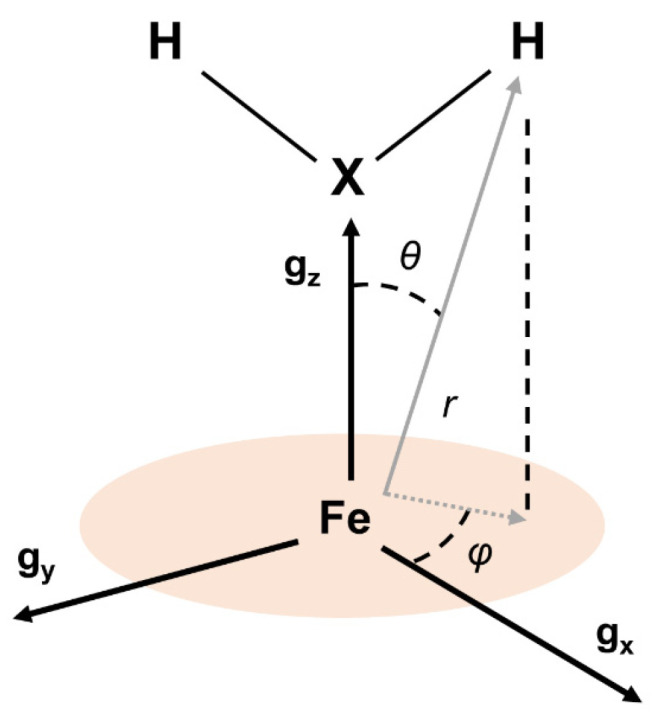
Sketch of Fe^III^-heme center showing the geometric relationships between the ***g***-tensor principal axes and a coupled proton nucleus belonging to an axial ligand and bound to a coordinated atom X.

The equation above is the expression of the hyperfine coupling between a nuclear and an electron spin with *g_N_* and *g_e_*, respectively. The distance between the two magnetic dipoles is represented by *r*, and *θ* is the angle between the vector $\vec{r}$ and the magnetic dipoles (oriented along the external magnetic field). For the HYSCORE spectra collected at *g_z_*, the magnetic field is oriented along the heme normal plane, thus *θ* is the angle between the heme normal plane and $\vec{r}$ (see Figure 2).

Starting from the structural data of the closest protons to the iron, obtained from the X-ray diffraction study of the protein [49] (in Table 2), the position of the HYSCORE signals was calculated assuming exclusively a dipole–dipole hyperfine interaction with the electron density concentrated entirely on the position of the iron ion. 

For cysteine beta protons (H^β^), the information about *r* and *θ* was obtained directly from the structure; for the water protons, a range of compatible distances and angles was estimated from the position of the distal water oxygen. This simple calculation was sufficient to preliminary assign H_1_ and H_2_ signals to the two axial water protons and H_3_ to one of the H^β^ of the cysteine proximal ligand since the calculations were already giving results very close to the experiment. The water proton assignment of H_1_ and H_2_ was further confirmed by the lack of corresponding signals in the HYSCORE spectrum of the protein recorded under the same conditions but in a deuterated buffer, together with the appearance of symmetric peaks above the ^2^H antidiagonal (see Figure 1b). The dipole–dipole couplings from other non-exchangeable protons in the active site, that is, the other H^β^ proton of the proximal cysteine and from the porphyrin, were calculated to be too small to have a contribution to the signal outside the diagonal peak, especially considering that porphyrin protons have been reported to contribute to ENDOR spectra at this field position with couplings of 2 MHz [50,51,52,53,54,55,56,57]. With this argument, we confirm the assignment of H_3_ signals to H^β^ of the proximal cysteine ligand.

The HYSCORE spectra at the other single-crystal position, *g_x_*, are depicted in the right column of Figure 1. Besides the matrix proton line at the diagonal, two short ridges are present along the ^1^H antidiagonal at frequency coordinates (17.0, 14.1) MHz, which slightly move and change shape upon buffer deuteration. Concomitantly, a strong peak at the diagonal, close to the Larmor frequency of the deuterium appears. In this case, the magnetic field of the molecules contributing to the spectrum lies on the heme plane, in the direction of the principal *x*-axis of the ***g***-tensor (which is in principle unknown). 

The spectrum for the intermediate position *g_y_* (central column in Figure 1) is contributed by molecules with different orientations of the magnetic field. The full range of orientations, containing the *y*-axis of the ***g***-tensor, is depicted in Figure S1 (see Supplementary Materials). In addition to a strong peak in the diagonal, four peaks symmetrically placed with respect to the diagonal and the ^1^H antidiagonal become evident together with weaker correlations forming a cross with the proton matrix peak at the center. These are combination peaks between the proton Larmor frequency (ν_H_) and the double quantum frequencies of ^14^N nuclei interacting with the electron spin (see below). Also, on the diagonal but slightly above the proton Larmor frequency, a small ridge is attributed to axial water protons based on their disappearance upon solvent deuteration (see Figure 1b, center).

**Figure 3:**
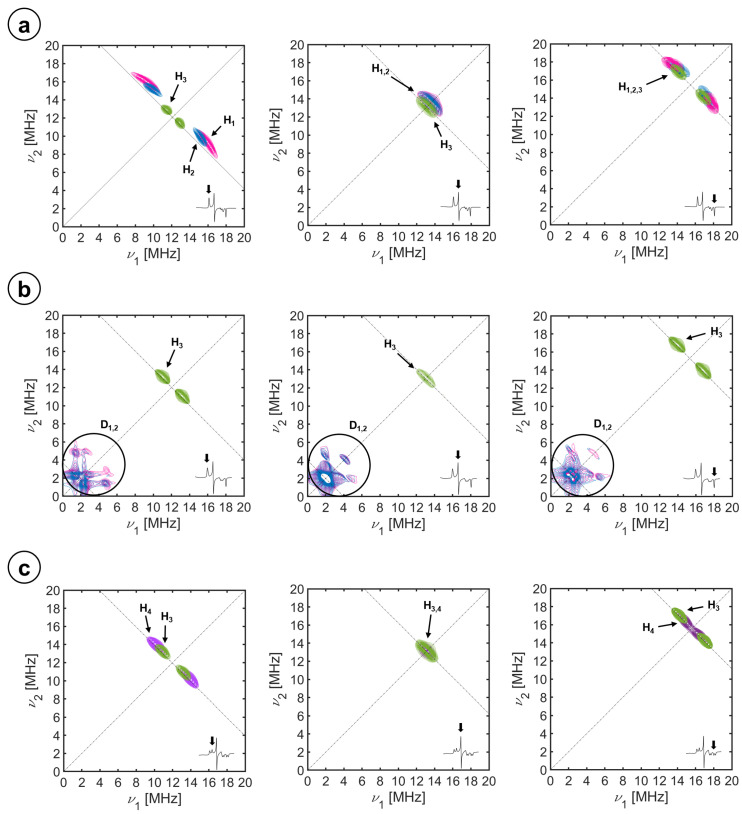
Simulation of the (+,+) quadrant, HYSCORE spectra of the substrate-free CYP116B5hd at the X-band. Exp. conditions: (300 μM) in KPi 50 mM pH 6.8, 30% glycerol in H_2_O (**a**), substrate-free in D_2_O (**b**), and interacting with ^15^N-imidazole (**c**). The spectra, as shown from the insets, were recorded at the (left column) *g*_z_, (center column) *g*_y_, and (right column) *g*_x_ magnetic field positions. The individual simulations of the water protons, H_1_ and H_2,_ are shown in pink and blue, those of the cysteine beta proton, H_3_, in green, and the imidazole protons, H_4_, in purple.

The first step of the analysis procedure after the described assignment was to process the data according to Dikanov’s procedure [58,59], as described in the Supplementary Materials. The correlation signals in the (ν^2^_α_, ν^2^_β_) plot are shown as straight lines, which indicates axial hyperfine interactions. Their linear fit allowed having a first estimate of the isotropic and axial anisotropic hyperfine contributions *T* and *a_iso_*. These values were subsequently refined using HYSCORE simulations (see Figure 3) whereby the orientation of the anisotropic hyperfine was also determined. 

The angles α and β are the two Euler angles connecting the gyromagnetic axes with the hyperfine frame. Since the hyperfine tensor is axial, there is no need for the third Euler angle, γ. The parameters yielding the best simulations for the ^1^H and ^2^H signals of the complete set of spectra (shown in Figure 3) are collected in Table 2. Note that the angle *β*, which is the angle between the normal plane to the heme (directed as *g*_z_ in our case) and $\vec{r}$ and *α*, which is the angle between the *g_x_* principal axis and the projection of $\vec{r}$ in the heme plane, corresponds to the angles *θ* and *φ* in Table 2 (see Figure 2). 

**Table 2:** Spin Hamiltonian parameters of ^1^H and ^2^H proton nuclei coupled to the Fe^III^ electron spin, derived from simulations in Figure 3 on the spectra reported in Figure 1.

Species	Label	*a_iso_* [MHz]	*T* [MHz]	EPR *α, β* [°]	EPR *r* (Fe-H) [Å]	*Crystal str.* *r* (Fe-H) [Å]	*Crystal str.* *θ* [°]	*Crystal str.* *φ* [°]
H_2_O	H_1_	−0.09 ± 0.06	5.60 ± 0.02	0 ± 5, 22 ± 5	2.42 ^a^	2.9 ^b^	23 ^b^	5 ^b^
H_2_O	H_2_	−1.095 ± 0.080	5.20 ± 0.02	0 ± 5, 16 ± 5	2.48 ^a^	2.7 ^b^	19 ^b^	0 ^b^
D_2_O	D_1_	−0.014 ± 0.01	0.860 ± 0.003	0 ± 5, 22 ± 5	2.42 ^a^			
D_2_O	D_2_	−0.17 ± 0.03	0.800 ± 0.003	0 ± 5, 16 ± 5	2.48 ^a^			
Cysteine	H_3_	0.79 ± 0.22	2.60 ± 0.04	0 ± 5, 47 ± 5	3.12 ^a^	3.078, 4.256 [49]	45, 66 ^c^	4, 13 ^c^
Imidazole (2)	H_4_	1.76 ± 0.11	2.66 ± 0.08	−25 ± 5, 40 ± 5	3.10 ^a^	3.142, 3.487 [60]	41, 38 ^e^	N.A.
Imidazole (1)		1.76 ± 0.11	2.66 ± 0.08	−60 ^d^, 40 ± 5				

^a^ Distances obtained through the point–dipole approximation (see Equation (1)). ^b^ Distances and angles obtained from the CYP116B5hd crystal structure (Res.: 2.60 Å) by tentatively adding the water protons using software. ^c^ Distances and angles obtained from the CYP116B5hd crystal structure, referring to the proximal cysteine, H^β^. The farthest proton is not resolved from the matrix line in the HYSCORE spectrum. ^d^ α angle obtained from the spectral simulation of ^14^N-Im *g_x_* HSYCORE features since no proton signals are observed at the *g_x_* of the Imidazole (1) species. ^e^ Distances and angles obtained from a CYP450_cam_—imidazole complex (Res.: 1.50 Å, see [60]) and referring to both imidazole protons, H(2) and H(5).

From the value of *T* obtained from the simulations, the iron–proton distance was estimated (column 6 in Table 2) and compared with the distance and *θ* angle obtained from X-ray diffraction experiments (column 7). The agreement is remarkable. Note that the best simulations are obtained for α = 0 for water and cysteine protons, which means the projection of the respective ***r*** vectors coincides with the axis *g_x_*.

The parameters for deuterium simulations were obtained by scaling the corresponding proton parameters by their nuclear gyromagnetic ratios. The simulations of the deuterium signals obtained using these hyperfine parameters together with a small nuclear quadrupole contribution of (0.1, −0.06, −0.04) MHz were satisfactory (see Figure 3). According to the crystal structure, the distance between the iron ion and the second H^β^ of the proximal cysteine is 4.26 Å so the expected value for *T* would be around 1.03 MHz. The heme meso protons are located at 4.50 Å from the iron center and the heme pyrrolic protons have distances between 5.88 Å and 7.74 Å, corresponding to *T* values of 0.87 MHz and 0.39 MHz and 0.17 MHz, respectively. Such couplings were not detected in the HYSCORE spectra; they probably remain unresolved from the proton matrix line. To obtain very weak couplings, Mims ENDOR was performed (see Figure S4). They showed unresolved couplings within 1 MHz of the proton Larmor frequency, none of which was attributable to an exchangeable proton.

Upon inhibition by imidazole, the HYSCORE patterns change. To maintain the focus on the proton signals, the spectra shown in Figure 1c correspond to the protein with an excess of ^15^N_2_-imidazole, which has a much weaker axial nitrogen nuclear modulation. In the *g_z_* spectrum, the proton signals assigned to the coordinated water molecule are absent in accordance with the replacement of the distal water ligand by the inhibitor. The proton signal that is actually detected consists of a ridge perpendicular to the diagonal. This signal overlaps with the correlation peaks detected in the substrate-free samples that were attributed to cysteine H^β^; however, there is a substantial difference in the shape of the signal, which is more elongated along the ^1^H antidiagonal. This difference is attributed to the additional contribution of 3- and 5-H imidazole protons. In the experimental spectrum at *g_x_* (Figure 1, bottom right), the cross peaks assigned to the cysteine protons that are clearly visible in the deuterated sample could not be detected. On the other hand, a relatively intense peak on the diagonal newly emerges. At *g_y_*, only a broad matrix peak on the diagonal is observed, which seems to be a bit more elongated along the diagonal than at the resting-state sample. The water proton signals vanished while the combination lines (*ν*_1H_ + ^14^N(*dq*)) remained. Simulations of the 3- and 5-imidazole protons flanking the coordinating nitrogen were performed using the point dipole approximation with Fe-N-Im distances from other imidazole-coordinated heme proteins as the starting point [60] to search for the α angle to optimize the fit. This optimum value was −25°, which defines the orientation of the imidazole plane with respect to the axis *g_x_*. The disappearance of the cysteine protons from the spectrum *g_x_* is puzzling and could be due to a change in the direction *g_x_* of the gyromagnetic tensor upon the addition of imidazole. We will investigate this instance carefully in the next section.

Note that the value of *a_iso_* found for imidazole protons (1.76 MHz) is larger than those from water (0.09 MHz and 1.095 MHz) and cysteine protons (0.79 MHz).

### 2.3. Hyperfine Interactions with Nitrogen Nuclei

**Figure 4:**
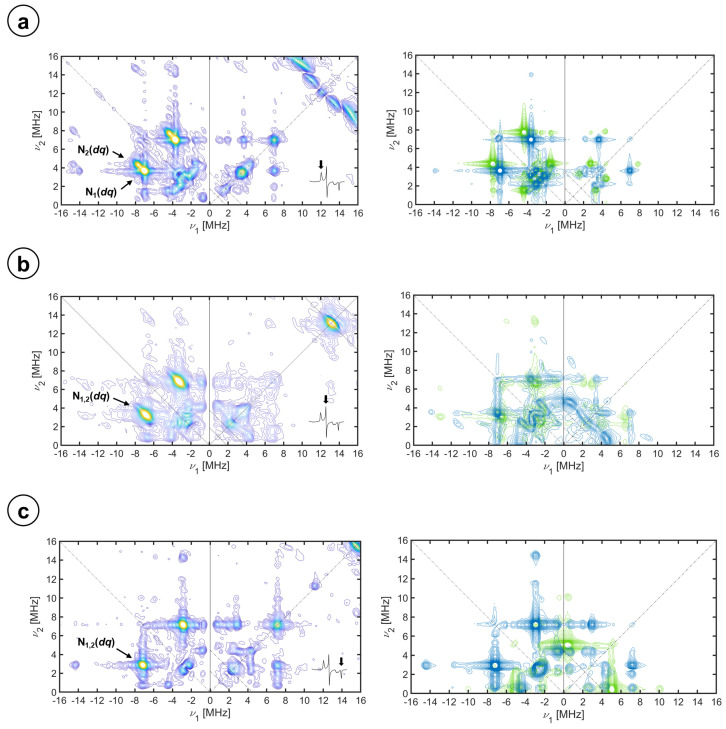
Experimental (**left**) and simulation (**right**) of X-band HYSCORE spectra of CYP116B5hd (300 μM) in KPi 50 mM pH 6.8, 30% glycerol substrate-free in H_2_O. The spectra were recorded at the (**a**) *g_z_*, (**b**) *g_y_*, and (**c**) *g_x_* magnetic field positions, at 10 K. τ values of (**a**) sum of 208 ns and 250 ns spectra, (**b**) 250 ns, and (**c**) 250 ns. The individual simulations of the heme ^14^N pairs, N_1_ and N_2_, are shown in green and blue, respectively.

In Figure 4, selected X-band HYSCORE spectra of CYP116B5hd in the H_2_O buffer showing the spectral regions with the contribution of ^14^N heme signals are displayed. 

In general, the spectra are dominated by strong signals in the (−, +) quadrant. In all cases, the position of the more intense features lies on the two lines parallel to the diagonal that intercept the coordinate axes at 4 *ν_N_*; that is, the difference between the nuclear frequencies in both spin manifolds is approximately four times the nuclear Larmor frequency of ^14^N at the given magnetic field. This allows assigning these features as correlations of *double quantum* (*dq*) nuclear frequencies of two different ^14^N nuclei (labeled as N_1_ (*dq*) and N_2_ (*dq*) in the figure) in the strong coupling regime (A > 2 *ν_N_*). For the spectra corresponding to *g*_z_, these peaks are found at (−7.00, 3.66) MH and (−7.61, 4.23) MHz, at (−6.77, 3.30) MHz for *g*_y_, and at (−7.14, 2.89) MHz for *g*_x_.

At frequencies below 5 MHz, there is a peak-dense region with correlation peaks involving *single quantum* (*sq*) nuclear frequencies that span both quadrants, (−,+) and (+,+).

The peaks observed at frequencies higher than 12 MHz in the (−,+) quadrant can be identified as combinations of nuclear frequencies of two nuclei. For the peak cluster around (−14, 4) MHz at the ***g***_z_ spectrum, one of the coordinates is the sum of *dq* frequencies in one spin manifold (2·*dq^N1^* and *dq^N1^* + *dq^N2^*) and the *dq^N1^* or *dq^N2^* nuclear frequency in the other spin manifold. Additional combination peaks are recognized above this cluster, for which the nuclear frequencies at both spin manifolds are the sum of *dq* frequencies. These combination peaks are also identified in the same region of spectra *g_y_* and *g_x_*, and they provide evidence of (at least) two equivalent nuclei of the one labeled N_1_(*dq*).

Further experiments were performed at higher microwave frequencies in order to increase resolution, an equivalent set of Q-band HYSCORE spectra is shown in the Supplementary Materials (see Figure S5). For the Q-band, the hyperfine coupling is not in the strong regime but rather it is close to the exact cancellation condition A~2 *ν_N_* for *g_z_* and, therefore, the correlations appear in both quadrants. For *g_x_* and *g_y_*, the correlation peaks are found in the (+,+) quadrant since the interaction is, for the corresponding magnetic field value, in the weak coupling regimen (A < 2 *ν_N_*). In the Q-band spectra, *dq* and *sq* correlations are observed, as labeled in the figure, but the s/n of the data did not allow recognition of combination peaks. 

The simulations that are shown in Figure 4 (right) and Figure S5 of the Supplementary Materials were conducted by fitting all the spectra with a common set of parameters. The simulation routines were started by trying to minimize the number of varied parameters; therefore, reasonable assumptions were made such as taking the heme normal plane (*g_z_*) as a principal axis for ***A*** and ***Q*** tensors of the nitrogen nuclei (see Equation (1)) [35,36,37,39], that is, the Euler angles for all tensors started as *β* = 0° and *γ* = 0°. In general, the hyperfine coupling parameters were estimated from the *dq* signals and the nuclear quadrupole couplings from the *sq* peaks. Taking the axes of the ***Q*** tensor of the heme ^14^N nuclei as the molecular axes, two groups of the ^14^N nuclei were considered that were initially bound to differ by 90° in their orientation of the nuclear quadrupole axes with respect to the ***g***-frame on the heme plane (Euler angle *α*). Complying with these assumptions, we were able to obtain the simulations shown in the figures without the need to release any of them. The simulation parameters are collected in Table 2. The hyperfine couplings of the two nitrogen nuclei differ only slightly, both hyperfine tensors are mostly isotropic, and the values are in the range of what has been reported before for other P450 enzymes and other low-spin heme centers [29,36,37,38,39,51,61]. While the isotropic contributions of the two sets of heme nitrogen nuclei are almost identical (N_1_, *a_iso_* = −4.93 MHz and N_2_, *a_iso_* = −5.10 MHz, see Section 3 for assignment of the sign), the anisotropic contributions are very small, a bit larger for the set N_2_ (N_1_, ***T*** = [0.035 0.135 −0.17] MHz and N_2_, ***T*** = [0.30 0.40 −0.70] MHz). The traceless nuclear quadrupole tensors have principal values similar to those that have been reported [29,36,39] and the orientation in the heme plane coincides approximately with the N-Fe-N directions.

**Figure 5:**
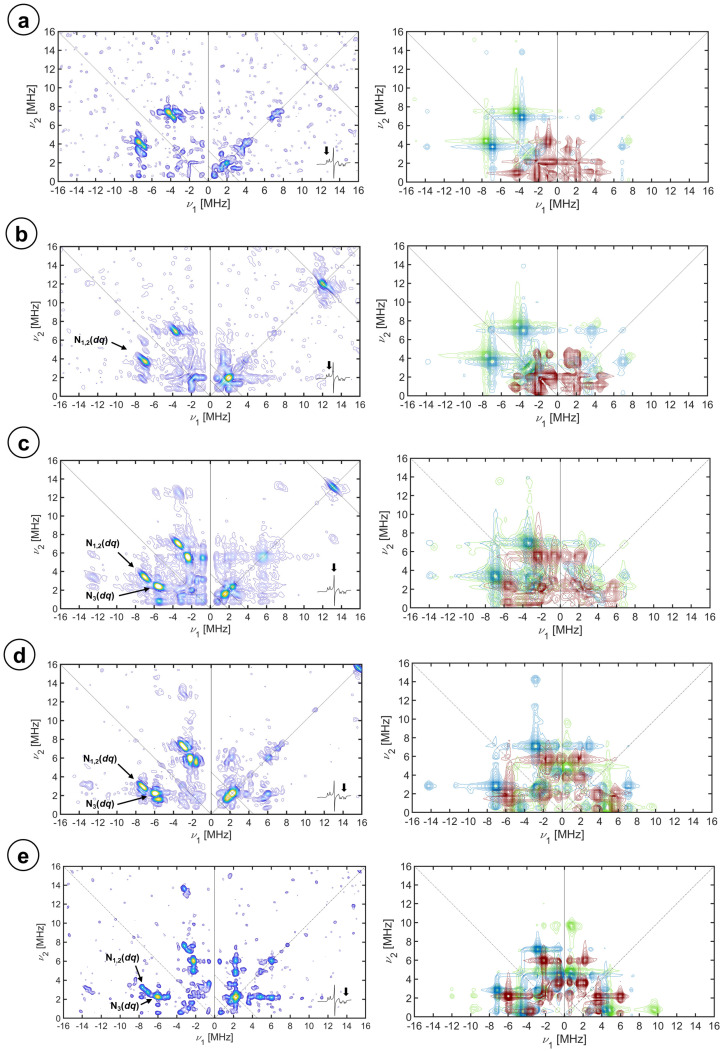
Experimental (**left**) and simulation (**right**) of X-band HYSCORE spectra of CYP116B5hd (300 μM) interacting with an excess of imidazole (1:10) in KPi 50 mM pH 6.8. The spectra were recorded at the magnetic field positions of (**a**) *g*_z_ (1), (**b**) *g*_z_ (2), (**c**) *g*_y_, (**d**) *g*_x_ (2), and (**e**) *g*_x_ (1) at 10 K. τ values of (**a**) 208 ns, (**b**) 250 ns, and (**c**) 250 ns. The individual simulations of the heme ^14^N pairs, N_1_ and N_2_, are shown in green and blue, respectively. The simulation of the imidazole ^14^N is shown in dark red.

Upon imidazole addition, as discussed above, the formation of two imidazole-bound species takes place. For the EPR spectrum of this mixture of species, only the positions *g_z_* and *g_x_* of the species labeled Imidazole (1), the one with the most anisotropic EPR spectrum, is in a single crystal-like position. The positions *g_z_* and *g_x_* of Imidazole (2), the least anisotropic species have a more intense echo signal, and the HYSCORE spectra, with better s/n, contain the contribution of a single orientation of this species plus a set of orientations of the most anisotropic species. The spectra are depicted in Figure 5. They clearly differ from the ones recorded at the equivalent field positions of the enzyme resting state in an aqueous buffer. We have previously identified ^14^N signals from the imidazole [32,33], but to make a complete assignment and analysis we prepared a sample by the addition of isotopically labeled ^15^N-imidazole to the resting state of the protein. The corresponding HYSCORE spectra are shown for both species in Figure S6 of the Supplementary Materials and are all very similar to the ones obtained for the aqueous resting state at equivalent magnetic field positions and are well reproduced with its parameters (collected in Table 2). Additional weaker ridges present in the spectra are attributed to the heme nitrogen atoms of the other (most anisotropic) imidazole-bound species. These results evidence the lack of a substantial perturbation of the iron electron density upon imidazole binding and rule out a change in the orientation of *g_x_*, which still coincides with one of the porphyrin N-Fe-N axes (α = 0). Also, a detailed comparison of the aqueous and ^15^N-imidazole bound spectra allowed the identification of weak ^15^N correlation peaks along the diagonal parallel crossing at 2·*ν*_15N_ for some of the spectra.

After studying the ^15^N-Im and ^14^N-heme signals, the signals from ^14^N-imidazole were analyzed using the spectra of protein samples prepared with an excess of naturally abundant imidazole, evidencing drastic changes in the patterns of the single quantum transitions, at frequencies below 5 MHz. The *dq* correlation peaks of a new strongly coupled nitrogen nucleus, labeled N_3_, at (−5.58, 2.40) MHz and (−5.96, 2.23) MHz frequencies at *g*_y_ and *g*_x_ magnetic field components of both Im-coordinated species are also visible in the spectra. The coupling tensors that best fit the imidazole ^14^N signals are collected in Table 3 and the corresponding simulations are shown in Figure 5 on the right. 

For both species, the ^14^N-Im hyperfine interaction was also found to be mostly isotropic (N_3_, *a_iso_* = −3.10 MHz) but somewhat weaker than the ones with the heme nitrogen nuclei. The small anisotropic contribution (N_3_, ***T*** = [−0.47 −0.10 0.56] MHz) is small, in the same range as that observed for the porphyrin nitrogen nuclei; but, in any case, the total hyperfine contribution is less than the one reported for coordinated nitrogen atoms of imidazole in bis-histidine or histidine-methionine coordinated heme systems [36,38,62]. The nuclear quadrupole values are, however, very similar to the ones reported for imidazole in these systems [36]. The quadrupole parameters of ^14^N-Im in many imidazole complexes have shown that the principal direction of a larger absolute value is the metal-^14^N bond (for us, *z*) and the smallest one corresponds to the normal imidazole plane [63]. Therefore, taking the ***Q***-tensor to indicate the orientation of the imidazole molecule, we determine that the orientation of the direction perpendicular to the imidazole plane with respect to the ***g***-frame, which does not seem to be affected by imidazole coordination, is about 65° for Imidazole (2) and 30° for Imidazole (1). This explains why the *g_z_* spectra are very similar for both species, whereas the *g_x_* spectra differ substantially.

## 3. Discussion

In the CYP116B5hd active site, the Fe^III^ is coordinated by the four nitrogen atoms of the heme ring, forming a very stable complex; in addition to that, a water molecule and a cysteine residue act as axial ligands. The octahedral geometry and the strong ligands coordinating the Fe^III^ define a low-spin electron state (*S* = ½), with the only unpaired electron of the ion dwelling in an orbital, which results from the mixing of the t_2g_ *d* orbitals caused by spin–orbit coupling [33,34,35,64,65]. Following this model, the ***g***-values obtained from the CW-EPR spectra of the enzyme and of low-spin hemeproteins, in general, can be related to the energy of the *t_2g_* orbitals of the iron considering a one-electron model (or rather a “hole”). The energy of the orbitals in a distorted octahedral environment can be parametrized by the axial crystal field parameter, ∆, and the rhombic crystal field parameter, V. Changes in the coordination environment of the Fe^III^-heme center are reflected in a change in the relative energy of the orbitals and a change in the crystal field parameters in units of the spin–orbit coupling constant, ξ. As expected, isotope labeling of the axial water or imidazole does not affect the coordination environment reflected in Δ and V, which is driven by electronic interactions. However, the replacement of the axial water by imidazole causes a decrease in the value of ∆/ξ, which indicates a change in the strength of the ligand field caused by the change in the axial ligand. Both imidazole-coordinated species have, within the error limits, the same ∆/ξ parameter. The V/ξ values, accounting for the lack of axiality in the spin system since it measures the energy difference between the *d_zx_* and *d_zy_* orbitals, are different for the two species, which, as we demonstrate here, is due to the imidazole plane adopting a different orientation with respect to the porphyrin ring [32,33].

The CW-EPR analysis (see Section 2.1) indeed helped to characterize the electron density distribution within the *t_2g_* orbitals and understand changes in the coordination geometry of the iron; however, the CW spectra do not allow studying the weak interactions between the iron unpaired electron spin and nuclei close by because the hyperfine structure is not resolved. Since further valuable information about the active site is contained in these interactions, the Fe^III^-heme system in the resting state and inhibited with imidazole was meticulously studied with HYSCORE, leading to the complete characterization of the hyperfine and nuclear quadrupole tensors of protons and nitrogen nuclei in the close environment of the electron spin. 

**Figure 6:**
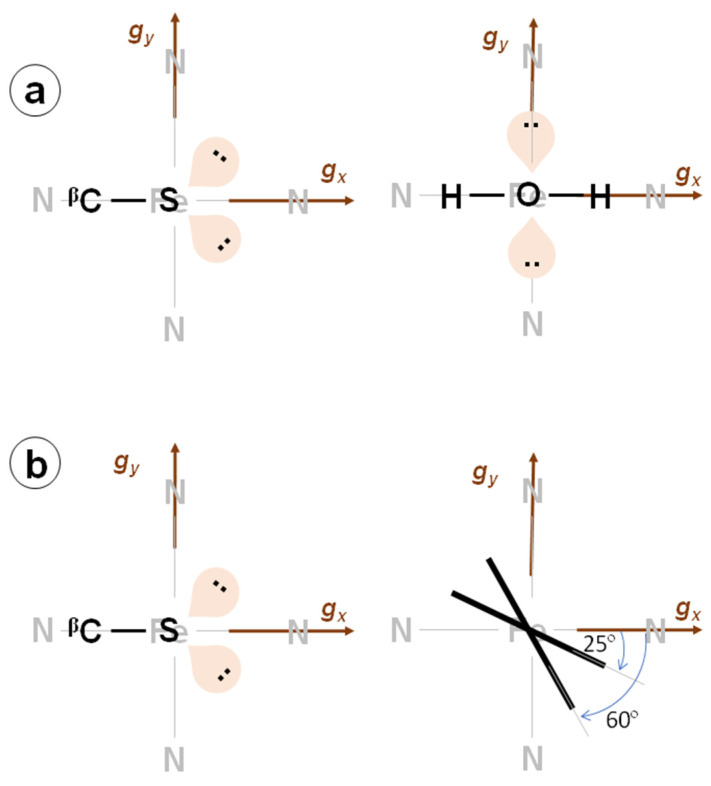
Sketch of the active site of CYP116B5hd. Views from the proximal (left) and distal sites (right). (**a**) CYP116B5hd in the resting state. (**b**) Imidazole inhibited CYP116B5.

The Dikanov methodology allowed us to determine that the proton hyperfine coupling tensors were axial and to obtain an estimation of *a_iso_* and *T* for the proton signals visible in the HYSCORE spectra that were later refined with simulations. From the value of the axial anisotropic hyperfine parameter *T* determined with this method and using the spin–nuclear dipole–dipole approximation (see Equation (2)), the distances between these nuclei and the unpaired electron of iron were calculated. These distances are highly consistent with the ones obtained from the crystal structure (see Table 1). Also, the Euler angle *β* was found to be very close to the angle *θ* measured between the heme normal plane and the vector $\vec{r}$ for every nucleus. This fact confirms that the principal axis associated with *g_z_* is directed along the heme normal plane. Additionally, the angle *φ*, which is the angle between the projection of the Fe-H bond onto the heme plane and the direction of *g**_x_***, was obtained for every proton by simulating the set of experimental data (this angle is the Euler angle α). The combination of *φ* values obtained for all protons allowed us to locate the direction of the *g**_x_*** axis itself, which coincides with the direction N (A pyrrole)-Fe-N (C pyrrole) of the heme plane. For the axial water molecule, we were able to resolve two different proton nuclei with very similar anisotropic couplings (~5 MHz), which is the main contribution to the hyperfine tensors for both protons but differs in the small isotropic hyperfine contribution *a*_iso_. The values of the distance estimated using the point dipole approximation are ~2.4 Å, which are consistent with what has been observed before for other CYP450s with a difference of 0.2 Å [52,66]. From the *α* angles of the hyperfine tensors, found to be 0 for both water protons, one can conclude that the plane of the water molecule is perpendicular to the heme plane lying on the *g_x_* axis. The distances and theta angles found for each of the two protons were very similar, which, taking into account that the two protons are in the same water molecule, could be interpreted as the coordination bond of Fe-O between the axial ligands and the iron by approximately bisecting the H-O-H angle (see Figure 6) as has been observed in previous studies [52,66]. 

The possibility of the two water protons belonging to two slightly different conformations of the axial water cannot be ruled out with the presented evidence; however, this result is not preferred since we think the extreme sensitivity of the CW-EPR spectrum to the conformation of the axial ligands would have shown two resolved species in the spectrum. As can be seen from Figure 1b, the presence of deuterated water in the active site is confirmed by the disappearance of the two external ridges (H_1_ and H_2_) from the proton signal pattern and the appearance of deuterium signals with hyperfine couplings that correspond perfectly to the reported values for the proton hyperfine tensors once the appropriate scaling is carried out according to their different nuclear Larmor frequencies. The nuclear quadrupole coupling observed is very small and consistent with what was observed before for water in a Fe-S cluster protein [67].

The signals from the cysteine β proton are consistent with those found for other CYP450s [30,40,66]. Again, the Fe-H^β-Cys^ distance obtained from the point dipole approximation of the hyperfine coupling and the tensor orientation allowed understanding that, from the two beta protons, the observed couplings originate from the one directed toward the heme, which is closer to the iron. Of course, the signals assigned to this proton do not disappear from the spectra upon solvent deuteration since only the water protons are exchangeable protons (pKa = 15.7); those of the cysteine, being an alkane-like species (pKa = 50), are not. The angle α obtained for this proton is 0 ± 5°, which indicates that the projection of the Fe-beta proton direction approximately coincides with *g_x_* (see Figure 6). Structural XRD studies of the protein show that this projection is 4° from the N (A pyrrole)-Fe-N (C pyrrole) direction. The cysteine proton signal that was observed in the *g_x_* spectrum should remain after the binding of imidazole unless the *g_x_* direction changes. We determined that this last hypothesis is not compatible with the observed nitrogen hyperfine structure; therefore, we conclude that for some unknown reason, we failed to detect these signals that lie below the s/n ratio of the spectra.

Parallel studies were carried out by means of the ENDOR technique, which is another useful method that is able to detect nuclei coupled to the unpaired electron; this method could have confirmed what was observed with HYSCORE. However, the results were unsatisfactory in detecting the near axial water molecule proton. The Davies ENDOR sequence yielded a very low s/n and hardly any signal was discernible. Regarding the distant heme peripheral protons (meso/pyrrolic) of the porphyrin ring, the Mims ENDOR showed only unresolved signals of non-exchangeable protons corresponding to couplings of less than 2 MHz.

The proton signals originating from the imidazole were never completely resolved from the ones of cysteine H^β^ in the HYSCORE spectra. The distances and *θ* angles obtained from the HYSCORE spectrum (note that there is no crystal structure of the Im-inhibited protein), correspond to what was seen before for axially coordinated imidazole cytochromes [60]. The *α* angle of 65° obtained for the species Imidazole (2) is interpreted as the orientation of the imidazole plane relative to the *g*_x_ direction (coinciding with the N_A_-Fe-N_C_ direction). The hyperfine couplings between the iron electron spin and the nitrogen nuclei also yield important structural information and further details about the electron spin localization in the active site of CYP116B5hd. In the resting state of the enzyme, the only nuclei strongly coupled to the metal are the ^14^N of the porphyrin covalently bound to the iron ion. The experimental spectra show two sets of non-equivalent nuclei that we call N_1_ and N_2_. This non-equivalency between the heme ^14^N has previously been observed in CYP450_cam_ or low-spin heme complexes [39], although they have been reported as equivalent in other ferric low-spin systems [29,62]. The various combination cross peaks emerging at the sum of *dq* nuclear frequencies that are detected in the different spectra led us to the conclusion that the signals of N_1_ originate from at least two magnetically equivalent nuclei.

The whole set of HYSCORE spectra at X- and Q-band frequencies could be reasonably simulated with the hypotheses that (1) the direction perpendicular to the porphyrin plane, as a symmetry axis, is approximately a principal axis of the hyperfine and nuclear quadrupole tensors for ^14^N coordinated nuclei and (2) the two sets of nitrogen nuclei correspond to the two sets of diametrically opposed heme nitrogen atoms and therefore their Euler α angles differ by 90°. Table 3 collects the parameters used for the simulations of the experimental data sets of nuclear frequency patterns (both shown in Figure 4). For both sets of nitrogen nuclei, N_1_ and N_2_, the hyperfine tensor is predominantly isotropic with principal values that are in the range of those found for pyrrole nitrogen nuclei of other ferric heme proteins [31,38,39,40,41,53,63]. The observed lack of magnetic equivalence of the four heme ^14^N ligands is basically due to a small but measurable difference in the hyperfine coupling constant along the heme normal plane, which is a bit larger for N_2_. This effect must be due to the breaking of the heme symmetry by distortions affecting the heme. Indeed, in the crystal structure at room temperature, the heme is reported to suffer from a small ruffling distortion: (in absolute values) 0.30 for CYP116B5hd, 0.38 for P450_cam_, and 0.09 for human aromatase, calculated using the PyDISH online tool at https://pydish.bio.info.hiroshima-cu.ac.jp/, accessed on 18 January 2024 [68]. 

Nitrogen combination cross-peaks similar to the ones observed here have been observed in bis-histidine low-spin heme systems which, together with an additional combination peak between a coupled proton and a heme nitrogen, allowed us to assign in this system a negative sign to the hyperfine coupling of the ^14^N coordinated nuclei [36] (heme and imidazole in that case). Based on the similarities of the two systems, we tentatively assign a negative sign to the hyperfine coupling constants of heme nitrogen atoms in CYP116B5hd. 

The nuclear quadrupole tensors obtained from our data are close to the ones reported for similar proteins [36,39]. The fact that the Q-tensor principal values are very similar, with a *β* Euler angle of 0° and an α angle close to 0° for one of the nitrogen sets (N_2_) and 90° for the other (N_1_) set, supports the premise that both sets are chemically equivalent but their spatial arrangement in the heme plane are rotated by 90°. Taking into account that the principal axes of the nuclear quadrupole tensors follow the molecular symmetry directions at the nucleus and that the larger principal value for pyrrole heme nitrogen nuclei has been reported to be perpendicular to the Fe-N direction in the heme plane, we can assign the set N_1_ to the nitrogen atoms, whose N-Fe-N direction is aligned with the *g_x_* direction (α~90°), and the set N_2_ to the other set of heme nitrogen atoms, whose N-Fe-N would be aligned with *g_y_*. Therefore, from both the heme nitrogen and the cysteine H^β^ nuclear frequency data, we can conclude that the direction of the *g_x_* axis (and therefore the *g_y_* axis too) coincides with the experimental uncertainty with the N-Fe-N directions of the heme. According to the counter-rotation theory [35], this means that the *t_2g_* orbitals are also oriented along the heme axes. One of the structural asymmetries of the axial ligands that are expected to split the energy of the orbitals *d_zx_* and *d_zy_* is the orientation of the second lone pair of sulfur in the cysteine. In the crystal structure, the projection of this lone pair onto the heme plane also approximately coincides with one of the heme axes (4°). Moreover, according to the water proton data presented here (*α* ~ 0°), the axial water arrangement also follows the heme directions. This makes sense since the axial water is free to rotate to attain the minimal energy configuration. In conclusion, from our experimental data, it seems that the porphyrin N-Fe-N bonds and the cysteine command the orientation of the Fe^III^ electron density and the axial water accommodates it.

The addition of the inhibitor strongly changes the appearance of the nitrogen HYSCORE spectrum in all the magnetic field positions. This is due to the presence of an extra nitrogen nucleus, which directly and strongly coordinates with the metal center. The inhibition of the protein with imidazole isotopically labeled with ^15^N allows for studying the effect of imidazole on the hyperfine structure of heme nitrogen nuclei and cysteine protons since the echo modulation amplitude due to the axial ^15^N is very small and hardly visible in the spectra. The data show that imidazole coordination does not disturb the hyperfine couplings for any of the imidazole-coordinated species. The large echo modulation amplitude due to the imidazole ^14^N nuclei allows for identifying its cross-peaks in all HYSCORE spectra and leads to the determination of the full hyperfine and nuclear quadrupole coupling tensors of this new ^14^N nucleus (Table 3). The hyperfine values are smaller (in absolute value) than those found for the iron-binding bis-His or His-Met heme centers, [36] but agree with those reported for the aromatase–anastrozole complex [29], which has cysteine–azo coordination. Moreover, hyperfine values of about 2 MHz have been reported for the axial amine’s nitrogen in imidazole–heme–mercaptoethanol and in pyridine–heme–mercaptoethanol complexes [69,70]. 

Additionally, according to a ^14^N and ^2^H quadrupole double resonance study of imidazole and substituted derivatives, the largest value of the nuclear quadrupole tensor in absolute value, |*Q_z_*| = 1.1 MHz, is an indication that the coordination occurs via the N(3) of the imidazole ring [71]. The difference of this value with respect to the one reported, |*Q_z_*| = 1.61 MHz, is explained by the fact that the addition of a Lewis acid (Fe^III^) to an amine (imidazole) leads to a reduction in the electric field gradient at the nitrogen nucleus [72]. This value is consistent with nuclear quadrupolar tensors measured for the N(His) of the myoglobin–mercaptoethanol complex and metal-coordinated nitrogen nuclei in different ligands, including imidazole, studied via nuclear quadrupole resonance [63,72]. The important feature to be emphasized from these studies is that nuclear quadrupole values of the order of those measured here for imidazole ^14^N are associated with the lone pair nitrogen donor orbital defining the principal axis of the nuclear quadrupolar coupling tensor (*Q_z_*). This orbital is axially directed toward the empty iron *d_z_*^2^ orbital forming an s bond along the heme normal plane. Simulation of the HYSCORE spectra demonstrates that *Q_z_* is nearly aligned along *g_z_* (0° < *β* < 10°), indicating that the axial nitrogen ligand is directed close to the heme normal plane. The Euler angle alpha of the ^14^N-imidazole nuclear quadrupole tensor indicates the projection of the direction perpendicular to the imidazole plane onto the heme plane. The HYSCORE patterns show that this orientation is 65° for the least anisotropic imidazole-coordinated species (Imidazole (2)), which is in full agreement with what was found in analyzing the imidazole proton signals, where the alpha angle indicates the orientation of the projection of the imidazole plane onto the heme plane. On the other hand, the orientation perpendicular to the imidazole plane was found to be closer to 30° for the second species (Imidazole (1)), which substantiates the differences between both species as hypothesized based on the crystal field parameter V/ξ. No cysteine proton signals were found but, interestingly, the hyperfine parameters of the ^14^N-heme ligands remained the same for the resting state and Imidazole (2), which confirms that heme and cysteine control the electronic structure of the iron. 

## 4. Materials and Methods

**Cloning, expression, and purification of CYP116B5hd.** The construct used in this work was obtained by cloning the initial part of the gene of CYP116B5 (coding for the first 442 amino acids, the heme domain) between NdeI and EcoRI restriction sites in a pET-30a(+) vector with the insertion of a N-terminal 6xHis-tag [19]. Expression and purification of the protein were carried out as previously described in detail in [19]. Briefly, protein expression was carried out in *E. coli* BL21 (DE3) cells at 22–24 °C for 24 h in LB medium supplemented with 0.5 mM of δ-aminolevulinic acid (δ-Ala) and 100 μM of IPTG. For the purification process, the cells were resuspended and sonicated in 50 mM of KPi buffer, pH 6.8, supplemented with 100 mM of KCl, 1 mg/mL of lysozyme, 1% of Triton X-100 and 1 mM of PMSF (phenylmethylsulphonyl fluoride), and 1 mM of benzamidine. After ultracentrifugation at 90,000× *g* for 45 min at 4 °C, the supernatant was loaded onto a 1 mL His-trap HP column (GE Healthcare, Chicago, IL, USA) and eluted using a linear gradient of imidazole ranging from 20 to 200 mM. The purest fractions were then concentrated, loaded into a Superdex 200 size exclusion chromatography column (GE Healthcare, Chicago, IL, USA), and eluted using 50 mM of KPi buffer, pH 6.8, containing 200 mM of KCl. The purified protein was then concentrated and stored in 50 mM of KPi buffer, pH 6.8, containing 10% glycerol after buffer exchange via ultrafiltration using Amicon Ultra 30,000 MWCO devices (Merck, Darmstadt, Germany). Deuterated protein samples were prepared by exchanging the aqueous buffer with 50 mM of KPi buffer, pH 6.8, containing 10% glycerol, prepared in D2O. Protein concentration was estimated from the spectrum of the P450−CO complex upon reduction with sodium dithionite and CO bubbling, using an extinction coefficient of 91,000 M^−1^ cm^−1^ [73].

**Electron Paramagnetic Resonance.** All the protein samples in 50 mM of KPi buffer, pH 6.8, with 10% glycerol were mixed with 30% glycerol as a glassing agent to an approximate final protein concentration of 200 μM. The samples with imidazole (natural abundance, hereinafter called simply imidazole) or ^15^N_2_-imidazole were prepared adding an excess of this chemical to reach the ratio (1:10) with respect to the protein. X-band experiments were performed on a Bruker ELEXSYS E580 spectrometer (microwave frequency 9.68 GHz) equipped with a cylindrical dielectric cavity and a continuous helium gas flow cryostat from Oxford Inc. (Atlanta, GA, USA). Q-band Pulse EPR experiments were performed on a Bruker ELEXSYS E580 spectrometer (microwave frequency 34 GHz) equipped with a continuous helium gas flow cryostat from Oxford Inc. (Atlanta, GA, USA). The magnetic field was measured by means of a Bruker ER035M NMR gaussmeter (Bruker, Ettlingen, Germany). 

*Continuous-Wave EPR.* The experiments were performed on the Bruker ELEXSYS E580 X-band spectrometer at a temperature of 40 K. A microwave power of 0.31 mW, a modulation amplitude of 0.6 mT, and a modulation frequency of 100 KHz were used. 

*Hyperfine Sublevel Correlation* (HYSCORE) [74]. Pulse EPR experiments were performed at 10 K using the pulse sequence π/2-τ-π/2-t_1_-π-t_2_-π/2-τ-echo with microwave pulse lengths t_π/2_ = 16 ns and t_π_ = 16 ns. The time intervals *t_1_* and *t_2_* were varied in steps of 16, 24, or 48 ns. To avoid overlooking correlation peaks due to blind spot effects, the experiments were performed for several τ values between 96 ns and 400 ns. A four-step phase cycle was used to remove unwanted echoes [75]. The raw time traces were baseline corrected with a third-order polynomial, apodized with a Hamming window, and zero filled. After the two-dimensional Fourier transformation, the absolute value spectra were calculated and plotted in 2D vs the two frequency axes. 

*Mims Electron Nuclear Double Resonance* (ENDOR) [76]. ENDOR experiments were carried out with a Bruker ESP 380E spectrometer (X-band) equipped with an EN 4118X-MD4 Bruker resonator with the pulse sequence π/2-τ-π/2-T-π/2-τ-echo, with a π/2 pulse of 16 ns and a radiofrequency (rf) pulse of 10 μs length.

*Simulations*. CW-EPR and HYSCORE spectra were simulated using the EasySpin^®^ toolbox for MATLAB [77]. The time traces obtained from the HYSCORE simulations were processed like the experimental ones, baseline corrected, apodised, and 2D-FFTransformed.

The simulations of the electron–nuclear spin system were performed using a spin Hamiltonian containing the electron Zeeman (EZ) term, a nuclear Zeeman (NZ), hyperfine (HF), and if *I* > ½, a nuclear quadrupole (NQ) term for every magnetic nucleus interacting with the electron spin:

(2) $${\hat{H}}_{0}={\hat{H}}_{\mathrm{EZ}}+{{\hat{H}}_{\mathrm{NZ}}+\hat{H}}_{\mathrm{HF}}+{\hat{H}}_{\mathrm{NQ}}=\frac{{2\pi \;\mu }_{B}}{h}B_{0}^{T}\;g\vec{\hat{S}}+\sum_{i}{\vec{\hat{S}}}^{T}A_{i}\vec{{\hat{I}}_{i}}+\frac{{2\pi \;\mu }_{N}}{h}\sum_{i}{g_{N,i}\;B}_{0}^{T}\vec{\hat{I}}+\sum_{I_{i}\;>\;\frac{\;1}{2}}{{\vec{\hat{I}}}_{i}}^{T}{\;Q}_{i}{\vec{\hat{I}}}_{i}$$

where *μ_B_* is the Bohr magneton, *h* is the Planck constant, *B*_0_ is the applied external magnetic field, $\vec{S}$ (*S* = ½) and $\vec{I}$ are the electron and nuclear spin operators, and ***g***, ***A***, and ***Q*** are the gyromagnetic, hyperfine, and nuclear quadrupole tensors, respectively.

To have a complete Hamiltonian formulation, the summation *i* should be the overall magnetic nuclei coupled to the electron spin, four porphyrin ^14^Ns, N-Im for the Im-inhibited protein, and several protons. However, to spare computational time, each nucleus was simulated separately and once the individual parameters were optimized, simulations considering more than one nucleus were performed selectively, specifically for those indicated in the text, to check for combination frequencies.

## 5. Conclusions

In this work, we presented a detailed analysis, by means of multifrequency Pulse EPR spectroscopy, of the active site of the peroxygenase-like CYP450, CYP116B5hd, in which the electronic location of the semi-occupied orbital is determined with respect to the heme site geometry and linked to the geometry of the axial ligands. Since during the reaction cycle, this protein receives 1 + 1 electrons from the reductase domain/partner that end up in the axial molecular oxygen ligand of the iron, the location of the orbitals where the transferred electron is hosted is a relevant piece of information to help understand the catalysis of this CYP450. The complete spin Hamiltonian parameters describing such a center were obtained with high accuracy and are consistent with similar systems previously characterized. The results showed that the imidazole binding, easily detectable with the joint effort of CW and Pulse EPR spectroscopy, does not severely alter the electronic environment of the Fe^III^-heme system. Moreover, the inhibitor ring orientation can be obtained through its interaction with the unpaired electron. Since CYP450s are involved in the metabolism of xenobiotics such as anti-cancer drugs, the study of imidazole inhibition of CYP450 could be useful for the development of imidazole-containing drugs to be used as coadjuvants to prolong and increase the effect of cancer treatments. 

## Figures and Tables

**Table 1:** The ***g***-values and crystal field parameters of the CYP116B5hd Fe^III^-heme system in different experimental conditions.

Sample		*g*_z_	*g*_y_	*g*_z_	V/ξ	∆/ξ
CYP116B5hd in D_2_O		2.443 ± 0.005	2.253 ± 0.002	1.923 ± 0.002	4.74 ± 0.06	5.44 ± 0.18
CYP116B5hd in H_2_O [32]		2.440 ± 0.005	2.250 ± 0.002	1.920 ± 0.002	4.74 ± 0.06	5.44 ± 0.18
CYP116B5hd in H_2_O + ^15^N_2_-imidazole	(2)	2.466 ± 0.005	2.258 ± 0.002	1.902 ± 0.002	3.47 ± 0.03	5.11 ± 0.17
	(1)	2.589 ± 0.005	2.258 ± 0.002	1.857 ± 0.002	4.40 ± 0.05	5.13 ± 0.17
CYP116B5hd in H_2_O + Imidazole [32]	(2)	2.468 ± 0.005	2.258 ± 0.002	1.902 ± 0.002	3.50 ± 0.03	5.13 ± 0.17
	(1)	2.585 ± 0.005	2.258 ± 0.002	1.860 ± 0.002	4.39 ± 0.05	5.14 ± 0.17

**Table 3:** Spin Hamiltonian parameters of ^14^N and ^15^N nuclei coupled to the Fe^III^ electron spin derived from simulations in Figure 4 and Figure 5 of the experimental spectra reported in Figures S5 and S6.

Species	Label	*A_x_* [MHz]	*A_y_* [MHz]	*A_z_* [MHz]	*α, β, γ* [°]	*Q_x_* [MHz]	*Q_y_* [MHz]	*Q_z_* [MHz]	*α*′, *β*′, *γ*′ [°]
Heme	N_1_	−4.90 ± 0.1	−4.80 ± 0.1	−5.10 ± 0.1	90 ± 5, 0 ± 5, 0 ± 5	0.90 ± 0.10	−0.60 ± 0.10	−0.30 ± 0.10	90 ± 5, 0 ± 5, 0 ± 5
	N_2_	−4.80 ± 0.1	−4.70 ± 0.1	−5.80 ± 0.1	0 ± 5, 0 ± 5, 0 ± 5	1.00 ± 0.10	−0.60 ± 0.10	−0.40 ± 0.10	0 ± 5, 0 ± 5, 0 ± 5
Imidazole (2)	N_3_	−3.57 0.1	−3.20 ± 0.1	−2.54 ± 0.1	65 ± 5, 0 ± 5, 0 ± 5	0.30 ± 0.10	0.80 ± 0.10	−1.10 ± 0.10	65 ± 5, 0 ± 5, 0 ± 5
Imidazole (1)	N_3_	−3.57 0.1	−3.20 ± 0.1	−2.54 ± 0.1	30 ± 5, 0 ± 5, 0 ± 5	0.30 ± 0.10	0.80 ± 0.10	−1.10 ± 0.10	30 ± 5, 0 ± 5, 0 ± 5
Imidazole-^15^N_2_	N_4_	5.00 ± 0.1	4.48 ± 0.1	3.56 ± 0.1	65 ± 5, 0 ± 5, 0 ± 5	N/A	N/A	N/A	N/A

## Data Availability

Data can be found at https://zenodo.org/records/10535347 and https://zenodo.org/communities/paracat_community, accessed on 18 January 2024.

## Supplementary Materials

The following supporting information can be downloaded at: https://www.mdpi.com/article/10.3390/molecules29020518/s1, Figure S1: Experimental X-band CW-EPR spectra; Figure S2: X-band echo detected field sweep EPR; Figure S3: Dikanov Methodology for the analysis of 1H HYSCORE Spectra; Figure S4: Mims ENDOR spectra of CYP116B5hd in H_2_O and D_2_O; Figure S5: Q-band HYSCORE spectra of CYP116B5hd in H_2_O—Experiments and simulations; Figure S6: HYSCORE spectra of CYP116B5hd interacting with imidazole-^15^N_2_; Figure S7: Complete HYSCORE spectra of CYP116B5hd in H2O; Figure S8: Complete HYSCORE spectra of CYP116B5hd in D_2_O; Figure S9: Complete HYSCORE spectra of CYP116B5hd interacting with imidazole in H_2_O; and Figure S10: Complete HYSCORE spectra of CYP116B5hd interacting with imidazole-^15^N_2_ in H_2_O.
