# Supplementary material for: Heme Spin Distribution in the Substrate-Free and Inhibited Novel CYP116B5hd: A Multifrequency Hyperfine Sublevel Correlation (HYSCORE) Study
Source: Molecules. 2024 Jan 20;29(2):518. doi: 10.3390/molecules29020518 (PMC10819608; doi:10.3390/molecules29020518)
Supplement: Supplementary file 1 [file molecules-29-00518-s001.zip › molecuels-2784772 Supplementary materials.pdf]

ELECTRONIC SUPPLEMENTARY INFORMATION

Spin Distribution in the Active Site of the Substrate-Free and Inhibited Novel CYP116B5hd: A Multifrequency HYSCORE Study

Antonino Famulari,[a,b] Danilo Correddu,[c] Giovanna Di Nardo,[c] Gianfranco Gilardi,[c] George Mitrikas,[d] Mario Chiesa,[b] Inés García-Rubio[a,e,*]

[a]Departamento de Física de la Materia Condensada, Universidad de Zaragoza, Calle Pedro Cerbuna 12, 50009, Zaragoza, Spain;
[b]Department of Chemistry, University of Turin, Via Giuria 9, 10125, Torino, Italy;
[c]Department of Life Sciences and Systems Biology, University of Turin, Via Accademia Albertina, 13, 10123, Turin, Italy;
[d] Institute of Nanoscience and Nanotechnology, NCSR Demokritos, 15341 Athens, Greece;
[e] Instituto de Nanociencia y Materiales de Aragón (INMA), CSIC-Universidad de Zaragoza, Zaragoza, Spain.

* Corresponding author

1. CW- and Pulse EPR Spectra of CYP116B5hd

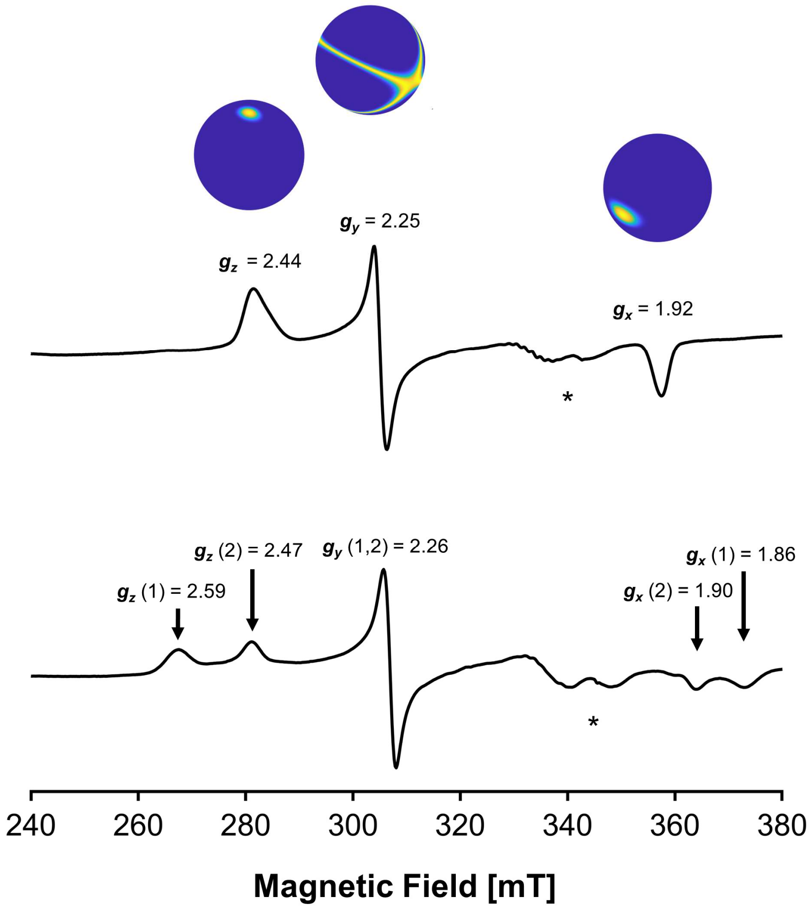

Figure S1. Experimental X-band CW-EPR spectra of a frozen solution of (top) CYP116B5hd, in the resting state, in deuterated buffer and (bottom) CYP116B5hd interacting with Imidazole-$^{15}N_2$. Buffer is 30 mM KPi pH 7.4, 30% glycerol. Both spectra were recorded at T = 40 K. The orientation selections on the unit spheres are shown for the observer position at g_z, g_y and g_x. * is a signal impurity.

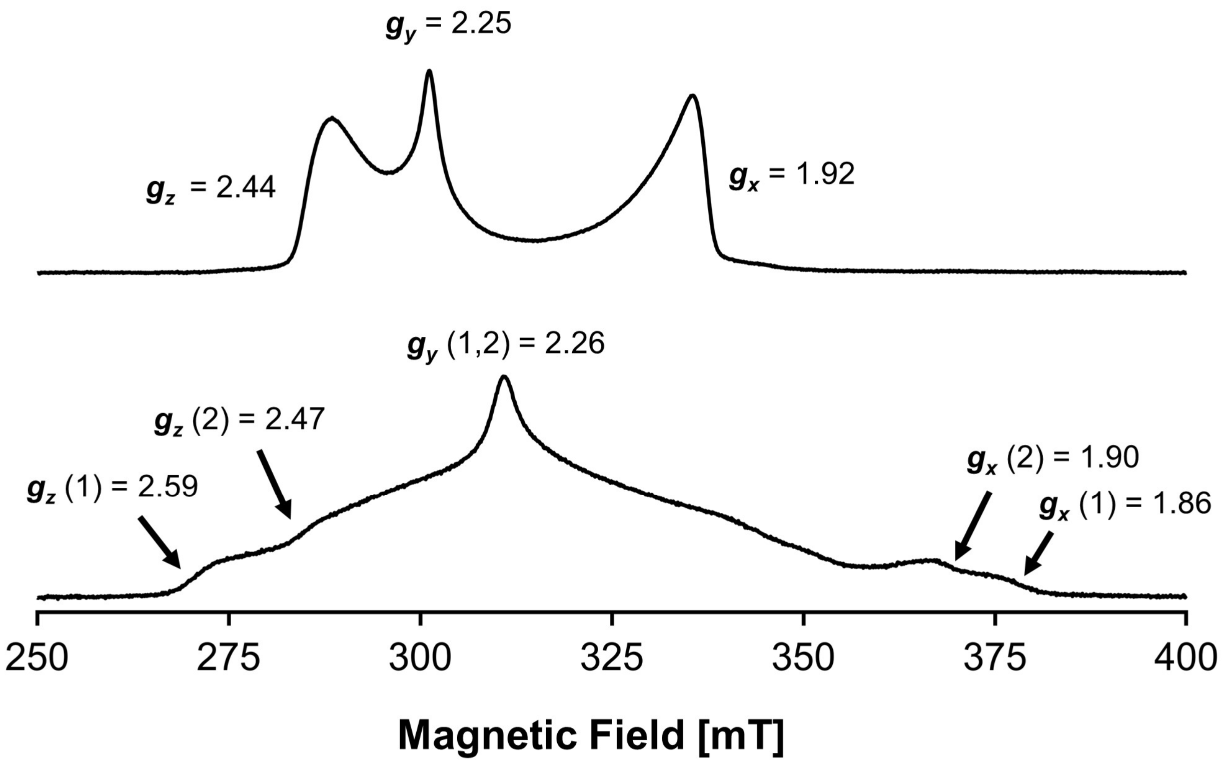

Figure S2. X-band echo detected field sweep EPR spectra of a frozen solution of (top) CYP116B5hd, in the resting state, in deuterated buffer and (bottom) CYP116B5hd interacting with Imidazole-$^{15}N_2$. The buffer is 30 mM KPi pH 7.4, 30% glycerol. Both spectra were recorded at $T = 10$ K.

2. Dikanov Methodology for the analysis of ^{1}H HYSCORE Spectra

The methodology created by Dikanov et al. [1] was used to analyze ^{1}H HYSCORE spectra since it enables the precise determination of isotropic and anisotropic hyperfine parameters without the need for comprehensive spectrum simulations. For an axial hyperfine interaction (HFI), with isotropic component $\boldsymbol{a}_{iso}$ and anisotropic tensor (−T, −T, 2T), i.e. $A = (A_x, A_y, A_z) = (\boldsymbol{a}_{iso} - T, \boldsymbol{a}_{iso} - T, \boldsymbol{a}_{iso} + 2T)$, the contour line shape in the powder 2D spectrum is described by [2]:

$$\nu_\beta = \left\{Q_\beta \nu_\alpha^2 + G_\beta\right\}^{1/2} \qquad (1)$$

with

$$Q_\beta = \frac{T+2a+4\nu_I}{T+2a-4\nu_I} \qquad (2)$$

and

$$G_\beta = -2\nu_I\left(\frac{4\nu_I^2-a^2+2T^2-aT}{T+2a-4\nu_I}\right) \qquad (3)$$

where ν_I is the nuclear Zeeman frequency. Note that these equations are valid for $\nu_\beta > \nu_a$. In the (ν_α, ν_β) plot, the shape of this cross-peak is an arc (see Figure S10a) but upon moving to a (ν^2_α, ν^2_β) plot it becomes a straight line with slope Q_β and intercept G_β (see Figure S10b). Performing a least square fitting of the (ν^2_α, ν^2_β) plot to a theoretical line, the slope and the intercept can be calculated and thus, two possible sets of the hyperfine coupling parameters $\boldsymbol{a}_{iso}$ and T can be obtained.

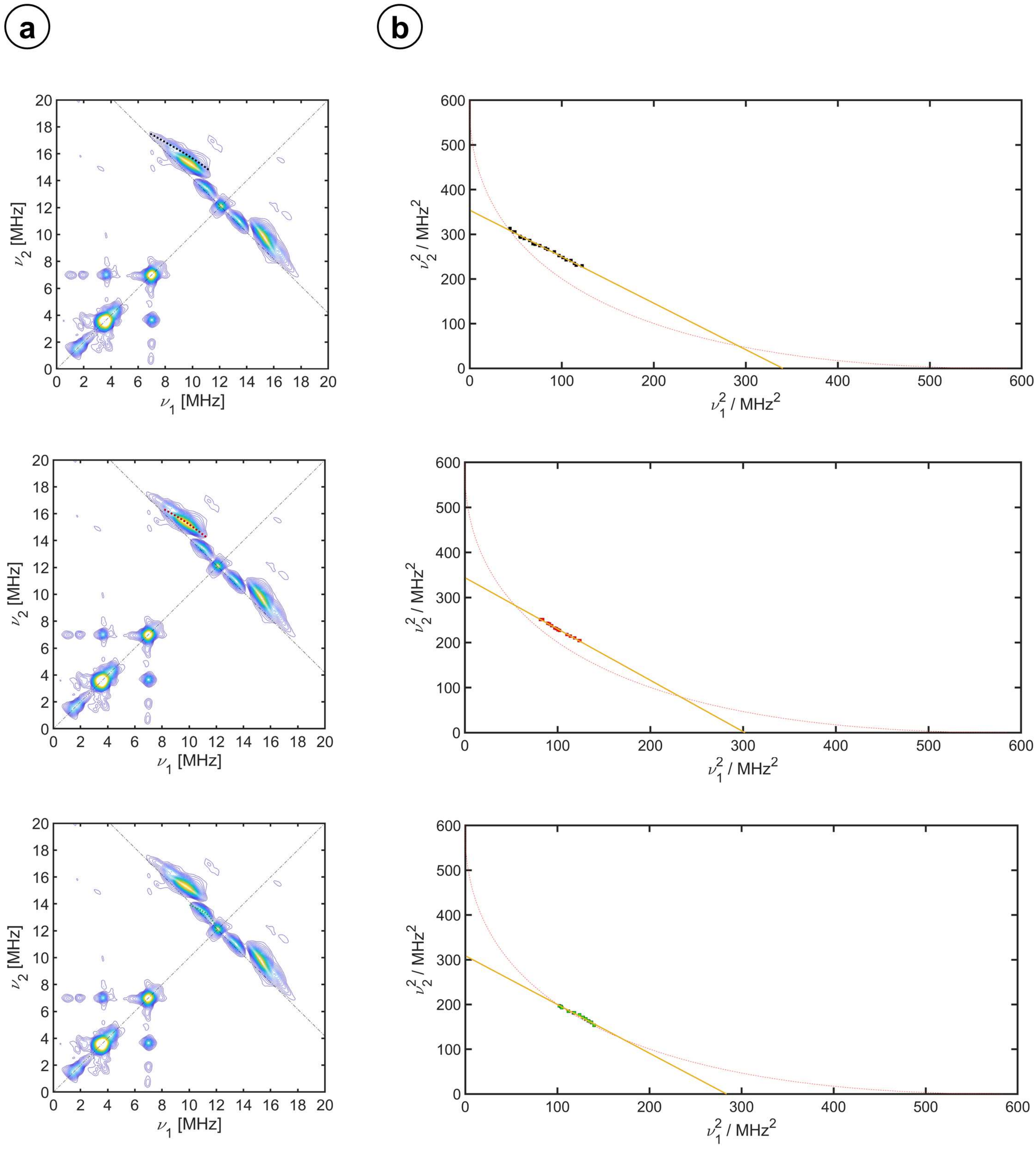

Figure S3. (a) ^{1}H HYSCORE spectrum of a frozen solution of CYP116B5hd in water with 30% of glycerol measured at g_z (B_0 = 283.8 mT). The superimposed symbols denote selected points of the three cross-peaks H_1 (black), H_2 (red), and H_3 (green) (check main text for the nomenclature). The antidiagonal line denotes the ^{1}H Larmor frequency, ν_H. (b) Points of cross-peaks in the ν_β^2 vs ν_α^2 representation of H_1 (top), H_2 (centre) and H_3 (bottom). The larger coordinate of each point is arbitrarily assigned to ν_β, and the smaller coordinate of each point is arbitrarily assigned to ν_α. The straight lines show linear fits of plotted data points using Equation 1. The dashed line is defined by the equation $|\nu_\alpha + \nu_\beta| = 2\nu_H$ with ν_H = 12.08 MHz. This figure is illustrative of the data processing of HYSCORE spectra, realized through the Dikanov methodology, of some proton nuclei signals found in the sample. The remaining proton nuclei signals, described in the main text, were processed in the same way but they are not shown for brevity's sake.

3. Mims ENDOR spectra of CYP116B5hd in H_2O and D_2O

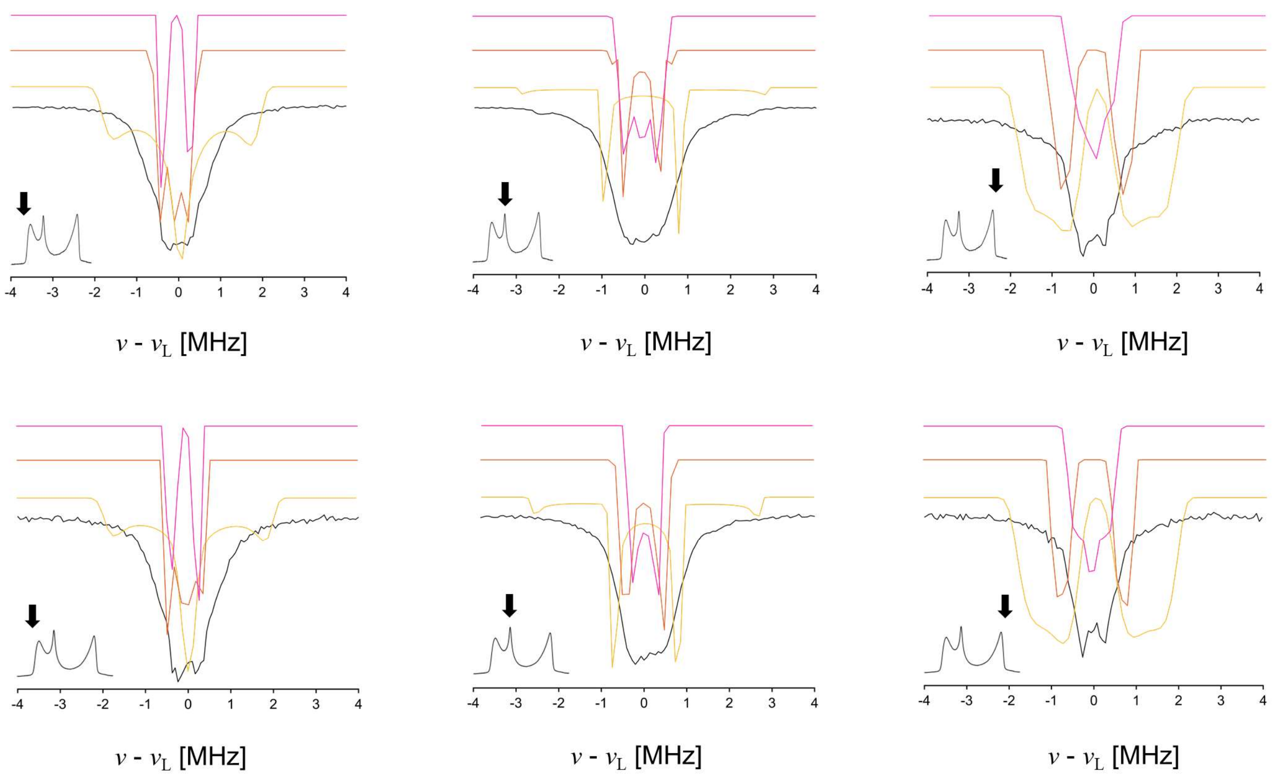

Figure S4. X-band experimental (black) Mims ^{1}H ENDOR spectra of CYP116B5hd and proton simulations (colour) in H_2O (top) and in D_2O (bottom) recorded at 9.45 GHz and 20 K. The spectra were recorded at the g_z (first column), g_y (second column) and g_x (third column) magnetic field positions. Each spectrum is the sum of nine spectra, recorded at the same magnetic field position, with τ values of 112 ns, 144 ns, 176 ns, 240 ns, 272 ns, 304 ns, 336 ns, 368 ns and 400 ns. The simulations correspond to three different sets of nuclei: one cysteine beta proton simulated with the same parameters obtained in this article and shown in main text Table 2 (yellow), one cysteine beta proton simulated with a_{iso} = 0.10 MHz and T = 1.02 MHz, α, β, γ = [13°, 66°, 0°] (brown) and four meso protons simulated with a_{iso} = 0.10 MHz and T = 0.87 MHz, α, β γ = [± 45°, 90°, 0°] (pink). The last two protons were simulated using distance (T) and geometry (angles) calculated from the crystal structure.

4. HYSCORE spectra of CYP116B5hd

This section contains HYSCORE spectra referred to in the main text, plus others shown in the main text or here but in a complete form.

2.1 Q-band HYSCORE spectra of CYP116B5hd in H_2O

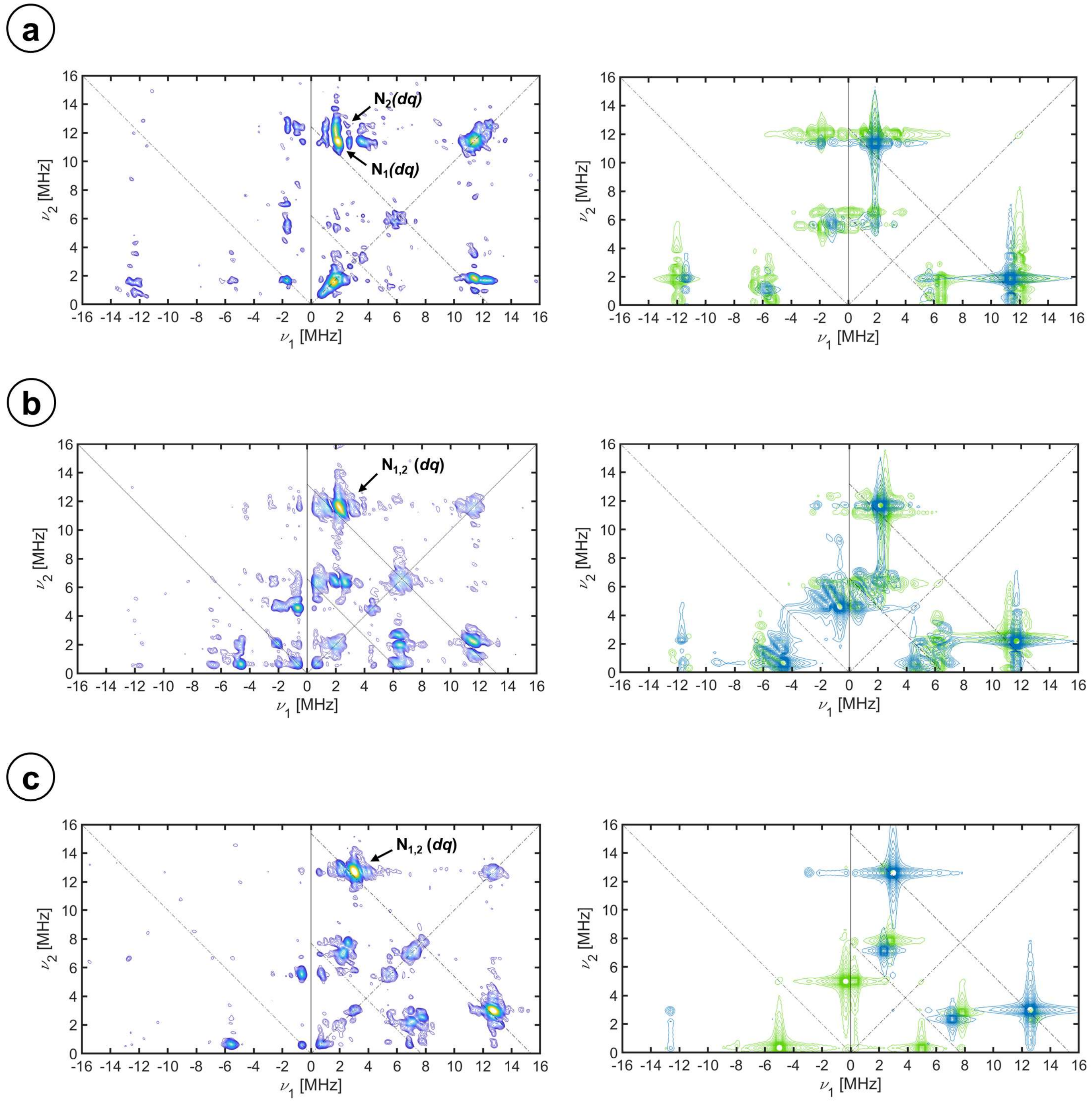

Figure S5. Experimental (left) and simulation (right) of Q-band HYSCORE spectra of CYP116B5hd (300 μM) in KPi 50 mM pH 6.8, 30% glycerol substrate free in H_2O. The spectra were recorded at the (a) g_z, (b) g_y and (c) g_x magnetic field positions, at 10 K. τ values of (a) 110 ns, (b) sum of 110 ns and 132 ns spectra and (c) sum of 132 ns and 172 ns spectra. The individual simulations of heme nitrogens, N_1 and N_2, are shown in blue and green respectively.

2.2 HYSCORE spectra of CYP116B5hd interacting with imidazole-$^{15}N_2$ in H_2O

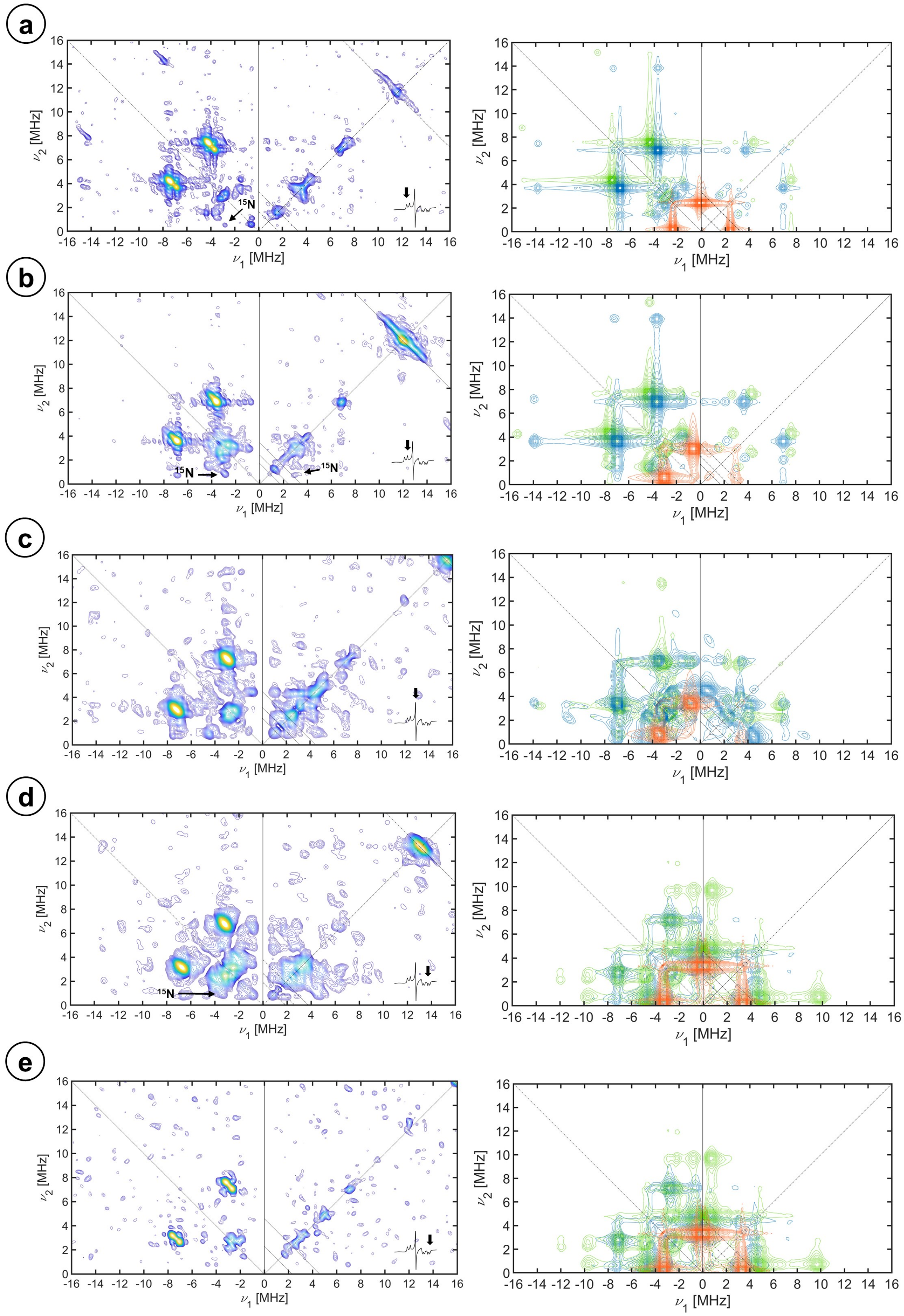

Figure S6. Experimental (left) and simulation (right) of X-band HYSCORE spectra of CYP116B5hd (300 μM) interacting with an excess of imidazole-$^{15}N_2$ in KPi 50 mM pH 6.8, 30% glycerol in H_2O. The spectra were recorded at the magnetic field positions (a) g_z (1), (b) g_z (2), (c) g_y and (d) g_x (2) and (e) g_x (1), at T = 10 K. τ values of (a) sum of 96 ns and 176 ns spectra, (b) sum of 208 ns, 250 ns and 400 ns spectra, (c) 250 ns, (d) sum of 250 ns and 400 ns, (e) sum of 208 ns and 400 ns. The individual simulations of heme ^{14}N, N_1 and N_2, are shown in green and blue respectively, while those of imidazole ^{15}N, N_4, in orange.

2.3 Complete HYSCORE spectra of CYP116B5hd in H_2O

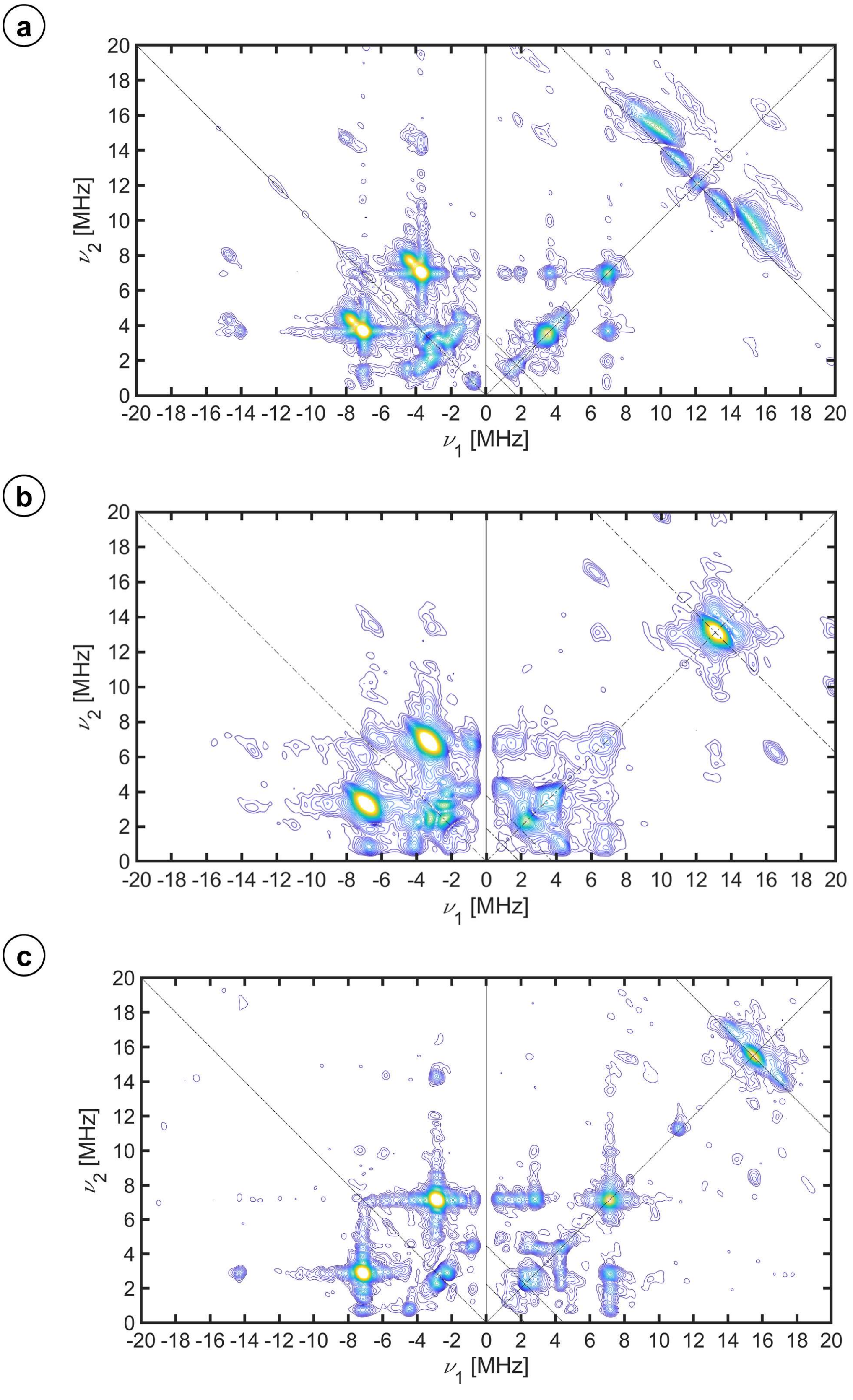

Figure S7. X-band HYSCORE spectra of CYP116B5hd (300 μM) in KPi 50 mM pH 6.8, 30% glycerol substrate free in H_2O. The spectra were recorded at the (a) g_z, (b) g_y and (c) g_x magnetic field positions, at 10 K. τ values of (a) sum of 208 ns and 250 ns spectra, (b) 250 ns and (c) 250 ns.

2.4 Complete HYSCORE spectra of CYP116B5hd in D_2O

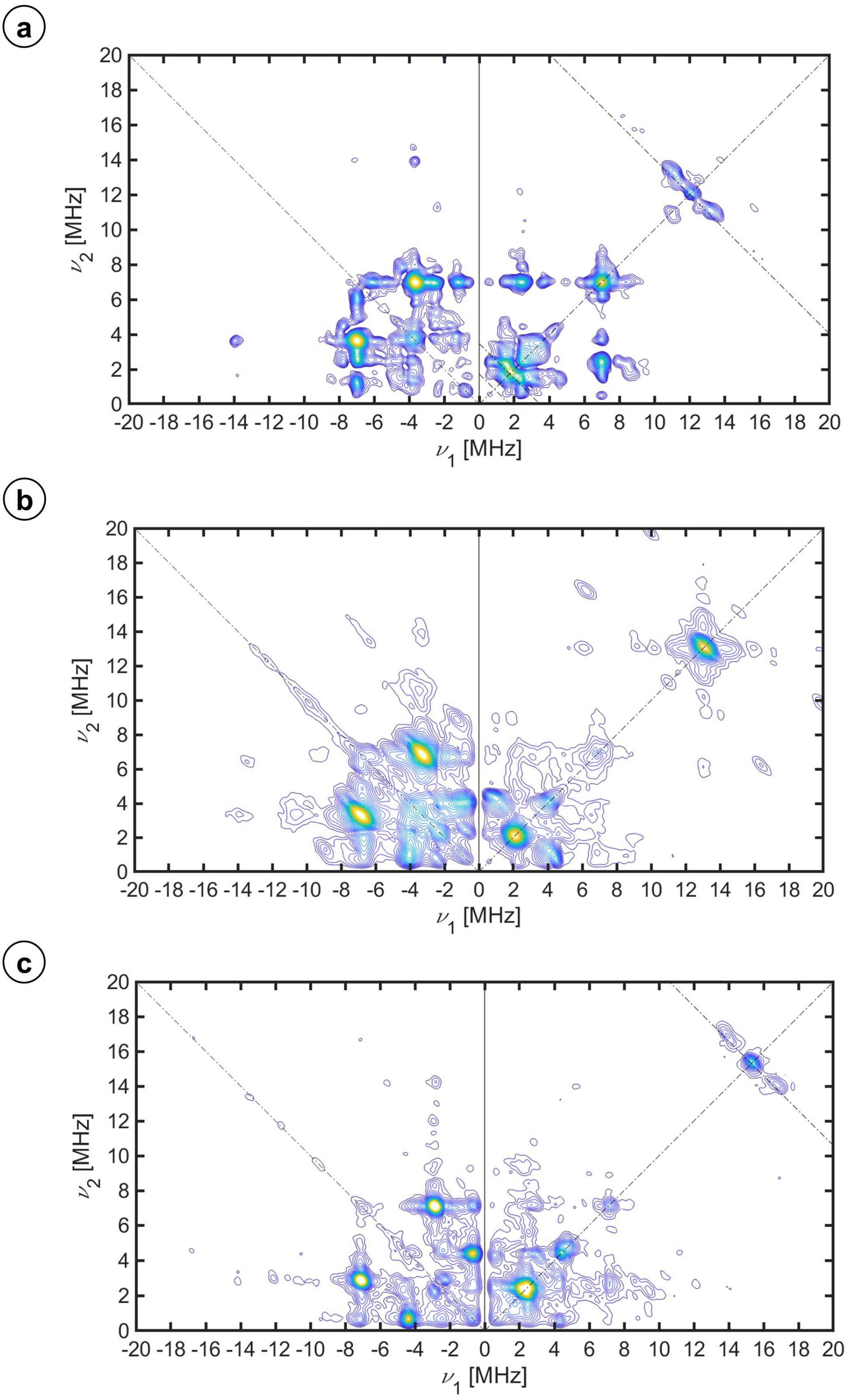

Figure S8. X-band HYSCORE spectra of CYP116B5hd (300 μM) in KPi 50 mM pH 6.8, 30% glycerol substrate free in D_2O. The spectra were recorded at the (a) g_z, (b) g_y and (c) g_x magnetic field positions, at 10 K. τ values of (a) 400 ns, (b) 250 ns and (c) 250 ns.

2.5 Complete HYSCORE spectra of CYP116B5hd interacting with imidazole in H_2O

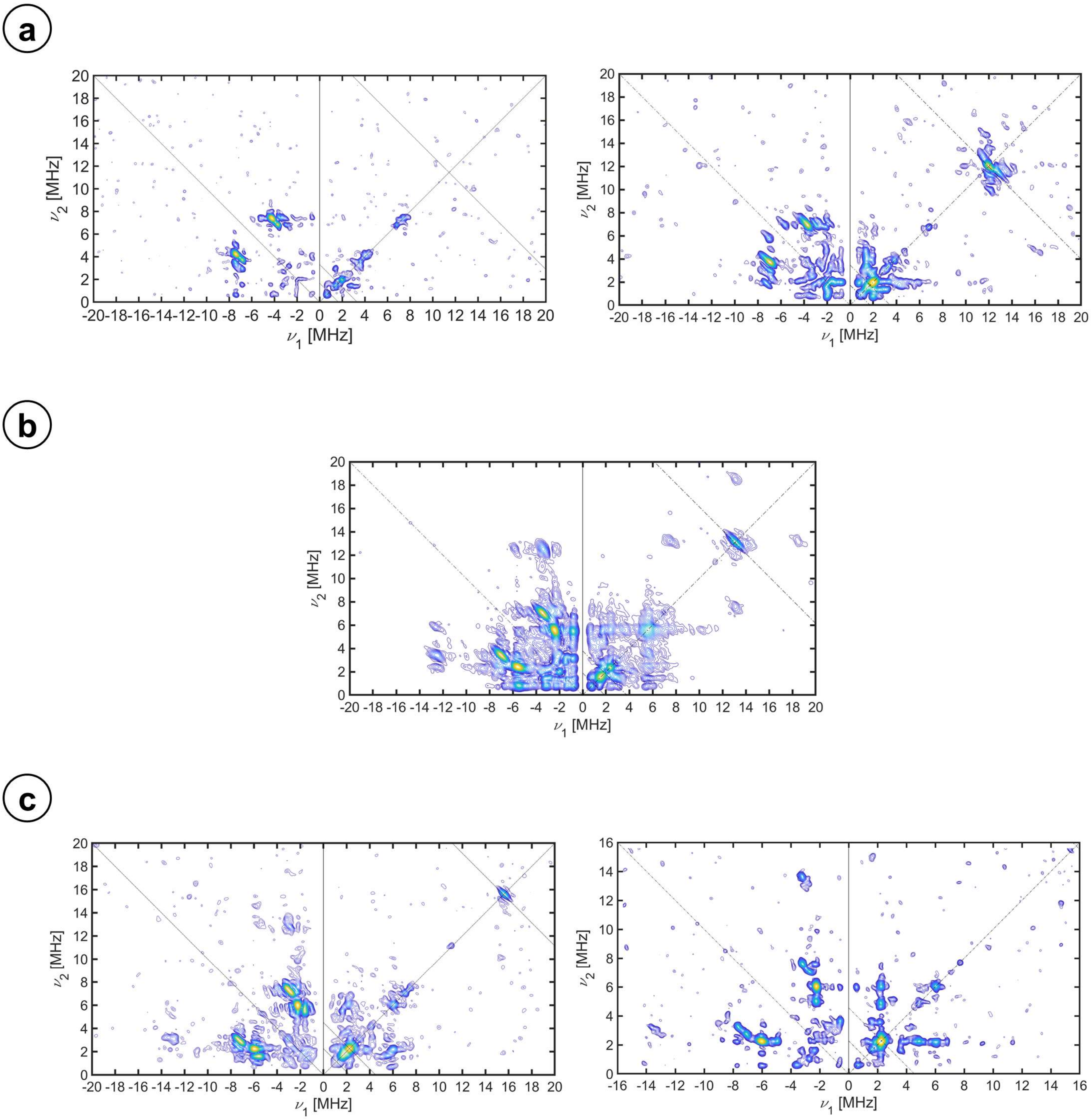

Figure S9. X-band HYSCORE spectra of CYP116B5hd (300 μM) interacting with an excess of imidazole (1:10) in KPi 50 mM pH 6.8, 30% glycerol substrate free in H_2O. The spectra were recorded at the magnetic field positions of (a) left: g_z (1), right: g_z (2); (b) g_y; (c) left: g_x (2), right: g_x (1). T = 10 K. τ values of (a), left, sum of 96 ns and 176 ns ns, (a), right, 208 ns, (b) sum 168 ns, 208 ns and 250 ns and (c), left, 250 ns (c), right, sum of 176 ns and 400 ns.

2.6 Complete HYSCORE spectra of CYP116B5hd interacting with imidazole-$^{15}N_2$ in H_2O

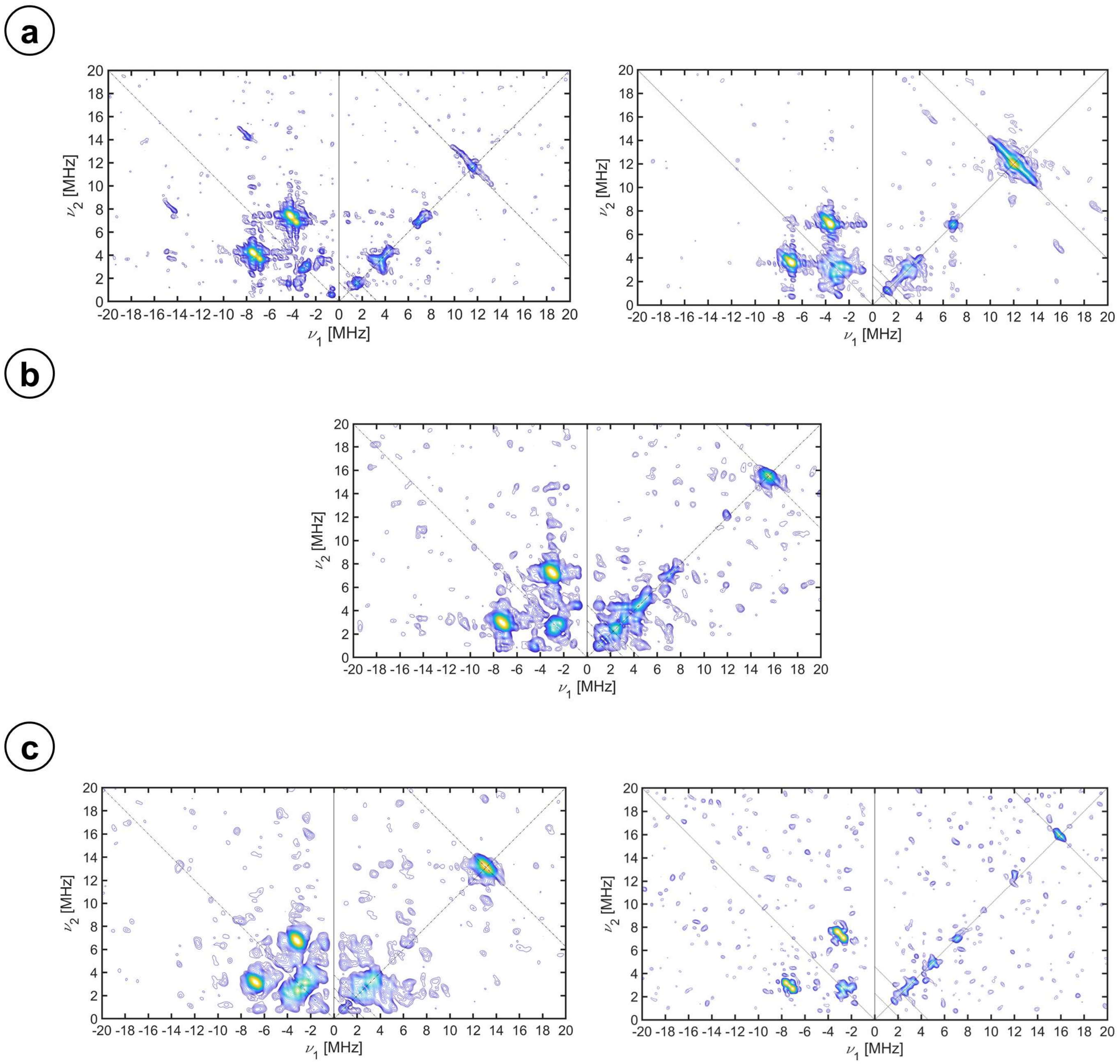

Figure S10. X-band HYSCORE spectra of CYP116B5hd (300 µM) interacting with an excess of ^{15}N-imidazole (1:10) in KPi 50 mM pH 6.8, 30% glycerol substrate free in H_2O. The spectra were recorded at the magnetic field positions (a) left: $g_z(1)$, right: $g_z(2)$; (b) g_y; (c) left: g_x (2), right: g_x (1). T = 10 K. τ values of (a), left, sum of 96 ns and 176 ns spectra, (a), right, sum of 208 ns, 250 ns and 400 ns spectra, (b) 250 ns and (c), left, sum of 250 ns and 400 ns (c), right, sum of 208 ns and 400 ns.

References

1. Dikanov, S.A.; Tyryshkin, A.M.; Bowman, M.K. Intensity of Cross-Peaks in Hyscore Spectra of S = 1/2, *I* = 1/2 Spin Systems. *Journal of Magnetic Resonance* **2000**, *144*, 228–242, doi:10.1006/jmre.2000.2055.
2. Dikanov, S.A.; Bowman, M.K. Cross-Peak Lineshape of Two-Dimensional ESEEM Spectra in Disordered S = 1/2, I = 1/2 Spin Systems. *J Magn Reson A* 1995, *116*, 125–128.
